# Integrating metabolomics, bionics, and culturomics to study probiotics-driven drug metabolism

**DOI:** 10.3389/fphar.2023.1047863

**Published:** 2023-01-26

**Authors:** Bohai Li, Lai-Yu Kwok, Dandan Wang, Lu Li, Shuai Guo, Yongfu Chen

**Affiliations:** ^1^ Key Laboratory of Dairy Biotechnology and Engineering, Ministry of Education, Inner Mongolia Agricultural University, Hohhot, Inner Mongolia, China; ^2^ Inner Mongolia Key Laboratory of Dairy Biotechnology and Engineering, Inner Mongolia Agricultural University, Hohhot, Inner Mongolia, China; ^3^ Key Laboratory of Dairy Products Processing, Ministry of Agriculture and Rural Affairs, Inner Mongolia Agricultural University, Hohhot, Inner Mongolia, China

**Keywords:** drug metabolism, probiotics, metabolomics, culturomics, bionics

## Abstract

Many drugs have been shown to be metabolized by the human gut microbiome, but probiotic-driven drug-metabolizing capacity is rarely explored. Here, we developed an integrated metabolomics, culturomics, and bionics framework for systematically studying probiotics-driven drug metabolism. We discovered that 75% (27/36 of the assayed drugs) were metabolized by five selected probiotics, and drugs containing nitro or azo groups were more readily metabolized. As proof-of-principle experiments, we showed that *Lacticaseibacillus casei* Zhang (LCZ) could metabolize racecadotril to its active products, S-acetylthiorphan and thiorphan, in monoculture, in a near-real simulated human digestion system, and in an *ex vivo* fecal co-culture system. However, a personalized effect was observed in the racecadotril-metabolizing activity of *L. casei* Zhang, depending on the individual’s host gut microbiome composition. Based on data generated by our workflow, we proposed a possible mechanism of interactions among *L. casei* Zhang, racecadotril, and host gut microbiome, providing practical guidance for probiotic-drug co-treatment and novel insights into precision probiotics.

## 1 Introduction

Aging, along with physical inactivity, overnutrition, and obesity, are major risk factors for chronic diseases, which have become a legitimate public health issue that limits healthspan (Franceschi et al., 2018). An increasing number of the global population suffers from cardiometabolic diseases, such as type 2 diabetes, obesity, hyperlipidemia, hypertension, and coronary artery disease, which oblige patients to take multiple drugs every day for months or even years (Ndisang and Rastogi, 2013). However, it has been widely recognized that the efficacy and toxicity of many drugs vary considerably between individuals. The typical response rates for commercially available drugs have been reported to be between 50%–75%, suggesting that on-sale drugs may not be effective in a large population of patients (Spear et al., 2001). Besides, severe drug side effects have killed more than 1,00,000 people and cost $30–100 billion per year in the United States (Sultana et al., 2013). Thus, inter-individual variability in drug responsiveness is a valid concern that not only delays treatment, posing uncertain impacts on patients’ health, but also causes enormous clinical and financial burdens to patients and society. Thus, it would be of significance to understand factors that result in the wide inter-individuality of drug responses.

The gut microbiota is a complex ecosystem that comprises trillions of cells, including bacteria, viruses, and fungi, and it encodes over three million genes, showing a broader metabolic potential than human cells (Kaoutari et al., 2013). The gut microbiota composition presents a high degree of inter-individual variation due to various environmental (e.g., diet, lifestyle, habits), physiological (e.g., age, health status), and genetic (e.g., ethnicity) factors (Falony et al., 2016). Growing evidence suggests that the gut microbiome is actively involved in drug metabolism. For example, Zimmermann et al. (2019a) systematically studied the interactions between drugs and microorganisms, reporting that 176 commonly used drugs could be metabolized by 76 human gut bacteria *via* chemical transformation (Zimmermann et al., 2019a). Various rodent and human studies have also shown that gut microbiota-mediated drug modifications could affect the bioavailability, bioactivity, and toxicity of drugs (Fu et al., 2015; Koppel et al., 2017; Zimmermann et al., 2019b). The large inter-individuality of human gut microbiota can thus contribute to the variation in drug responses, and it can be considered as a potential target for modulating drug efficacy and toxicity.

Probiotics are “live microorganisms which when administered in adequate amounts confer a health benefit on the host” (FAO/WHO, 2001). Driven by an increasing health consciousness of the general public, the trend of consuming probiotics is on the rise. The probiotics market size is predicted to reach around US$ 133.92 billion by 2030 from US$ 63.11 billion in 2021 (GlobeNewswire, 2022). Probiotics have gained wide popularity and are commonly added to various food and health products, e.g., yogurts, cheese, juices, nutrition bars, infant formulas, sweeteners, waters, pizza crust, gum, lozenges, dietary supplements, and so on (Sadiq et al., 2022). Moreover, probiotics have been increasingly applied in clinical settings for preventing and treating multiple medical conditions, partly due to their capacity to restore a healthier gut microbiota from disease-associated gut dysbiotic state (Gagliardi et al., 2018). Like other gut microorganisms, probiotics encode a variety of enzymes and have been demonstrated to metabolize various chemicals, such as organophosphorus pesticides, mycotoxins, and nitrosamines in both vitro and vivo studies (Średnicka et al., 2021). Thus, the wide application of probiotics as active food components can also influence drug efficacy and side effects. However, data on probiotic-drug interactions are very scant.

This work comprises a series of experiments aiming to assay probiotic-drug interactions systematically by: 1) profiling the capacity of five selected probiotic strains [namely, *Bifidobacterium animalis* subsp. lactis Probio-M8 (M8), *B. animalis* subsp. lactis V9 (V9), *Lactiplantibacillus plantarum* P8 (P8), *Lacticaseibacillus rhamnosus* Probio-M9 (M9), and *Lacticaseibacillus casei* Zhang (LCZ)] in degrading 36 commonly used clinical oral drugs; 2) further characterizing the ability of LCZ in metabolizing racecadotril into its active forms in monoculture, in a near-real simulator human digestion system, and as part of the *ex vivo* human fecal culture. Our work demonstrated the ability of probiotics to degrade commonly used drugs through their own metabolism and/or *via* modulating the gut microbiome in a selective manner. This study has provided a framework for studying probiotic-drug interactions and novel insights into probiotic-drug co-treatment and probiotic-based personalized therapy.

## 2 Materials and methods

### 2.1 Chemicals

All 36 investigated drugs and the two standard solutions (thiorphan and S-acetylthiorphan) were commercially purchased from Sigma-Aldrich (St. Louis, MO, United States), and the detailed information about these compounds is listed in Supplementary Table S1. Acetonitrile, methanol, dimethyl sulfoxide, formic acid, and ammonium acetate were HPLC grade, and along with KCl, KH_2_PO_4_, NaHCO_3_, NaCl, MgCl_2_(H2O)_6_, (NH_4_)_2_CO_3_, CaCl_2_(H_2_O)_2_, L-cysteine, ethyl acetate, HCl, and NaOH were bought from Merck (Darmstadt, Germany). Bryant and Burkey Medium (BB), de Man Rogosa Sharpe (MRS) broth, glycerol, human salivary α-amylase, porcine pepsin, rabbit gastric extract for gastric lipase, bovine bile, and porcine pancreatin were purchased from Sigma-Aldrich (St. Louis, MO, United States). Modified Gifu anaerobic medium (mGAM) broth was purchased from HyServe GmbH and Co., KG (Germany). Ultrapure water used throughout the study was prepared by a Milli-Q water purification system (Millipore, Molsheim, France).

### 2.2 Probiotic strains and culture conditions

Five probiotic strains were used in this study, including M8, V9, P8, M9, and LCZ. They were obtained from the Lactic Acid Bacteria Culture Collection (LABCC) of the Key Laboratory of Dairy Biotechnology and Engineering, Inner Mongolia Agricultural University. These five strains have been proven to possess favorable probiotic properties and are applied in food, beverage, silage, and other products.

The five bacterial strains were cultured anaerobically for 24 h at 37°C in the MRS medium with 0.5 g/L L-cysteine (ML medium). Each strain was subcultured twice in the ML medium before use.

### 2.3 Assaying the drug-metabolizing capacity of five different probiotic strains

#### 2.3.1 Sample preparation

In an anaerobic chamber (Don Whitley Scientific, Bingley, United Kingdom), each probiotic strain (adjusted to 5 × 10^6^ CFU/mL) was inoculated into 10 mL of ML medium. Then, 10 µL of each of the 36 tested drugs (dissolved in dimethyl sulfoxide, 1 mg/mL) was added to the probiotic-inoculated medium. A negative control was processed in parallel by adding 10 µL of each drug to sterile ML medium without bacterial inoculation. Cultures and controls were incubated under the same conditions (at 37°C) in the anaerobic chamber for 24 h. Afterward, cultures were extracted with 20 mL of ethyl acetate, and the organic phase was collected and concentrated using the Genevac EZ-2.3 ELITE centrifugal evaporator (SP Scientific, Inc., New York, United States). The residue was resuspended in 500 µL methanol and centrifuged at 13,000 g, 4°C for 10 min, and the supernatants were passed through 0.22 μm microporous membrane filters and were stored at −80°C for further liquid chromatography-mass spectrometry (LC-MS) analysis. The experiments were performed in triplicate.

#### 2.3.2 Standards and calibration

Stock solutions of 10 mg/mL concentration (dissolved in HPLC grade dimethyl sulfoxide) were prepared for all reference standards. Each stock standard solution was then subjected to serial dilution with sterile ML medium from a concentration range of 5 ng/mL to 1 μg/mL. The diluted stocks were extracted using the method described above and were stored at −80°C before LC-MS analysis.

#### 2.3.3 Targeted quantitative metabolomics analysis

Targeted quantitative metabolomics analysis was performed on an ultra high performance liquid chromatography coupled with triple quadrupole mass spectrometry (UPLC-QqQ-MS/MS) system (SCIEX Exion LC coupled to a SCIEX QTRAP 6500+; SCIEX, Foster City, CA, United States) along with a Kinetex EVO C18 column (2.1 mm × 100 mm, 1.7 μm, Phenomenex, California, Co, United States). The conditions of UPLC were as follows: autosampler compartment temperature, 4°C; column compartment temperature, 40°C; mobile phase A, ultrapure water containing 0.1% formic acid; mobile phase B, methanol containing 0.1% formic acid. The gradient elution was programmed as follows: 0.0–1.0 min, 5.0% B; 1.0–6.5 min, 5.0%–100.0% B; 6.5–9.5 min, 100.0%–100.0% B; 9.5–11.0 min, 100.0%–5.0% B; 11.0–12.0 min 5.0% B. The flow rate was 0.4 mL/min, and the injection volume was 1 μL. The parameters of mass spectrometry were set as follows: curtain gas, 25 psi; ion source gas 1, 50 psi; ion source gas 2, 50 psi; source temperature, 550°C; ion spray voltage in positive mode, 5,500 V; ion spray voltage in negative mode, −4,500 V; carrier gas, nitrogen. Multiple reaction monitoring was used for drug detection and quantification.

#### 2.3.4 UPLC-QqQ-MS/MS data analysis

Data were acquired by the Analyst 1.7.1 software and analyzed with the SCIEX OS-Q software (both supplied by SCIEX, Foster City, CA, United States), including standard curve construction and sample quantification. All further data processing was performed in R-4.1.2 (https://www.R-project.org/) after sample quantification. The drug degradation rate was calculated according to the following formula: degradation rate (%) = (R2-R1)/R2 × 100, where R1 and R2 represented the concentrations of drugs after 24 h incubation with and without inoculation with probiotics, respectively. The LC-MS system has an instrument-dependent random systematic error range of around 20%–30% (Chong et al., 2019). Thus, in this study, the degradation rate threshold was set at 30% to ensure that the detected drop in drug concentration was a specific probiotic metabolic effect. Wilcoxon tests (calculated by kruskal. test function in dplyr R package) were applied to test whether drug levels were significantly lower in the probiotics-drug conditions compared with the controls. All *p*-values were adjusted for multiple hypothesis testing with the Benjamini-Hochberg procedure (*p*.adjust function in graphics R package).

### 2.4 Identification of racecadotril metabolites produced by *in vitro* LCZ culture

#### 2.4.1 Growth curve construction

In the anaerobic chamber, 5 × 10^6^ CFU/mL of LCZ was inoculated into 10 mL of ML medium and incubated anaerobically at 37°C for 24 h. The growth curves of LCZ were determined by measuring the optical density of cultures at 600 nm (OD600) using a Bioscreen C system (Oy Growth Curves AB Ltd., Helsinki, Finland). The experiments were performed in triplicate.

#### 2.4.2 Sample preparation

In the anaerobic chamber, LCZ was inoculated at 5 × 10^6^ CFU/mL into 10 mL of ML medium. Then, 10 µL of racecadotril (10 mg/mL) was added to the ML medium with and without LCZ. Three groups were included in this experiment: LCZ-drug (LCZ was cultivated with racecadotril), LCZ-medium (LCZ was cultivated without racecadotril), and drug-medium (contained only racecadotril without bacteria). All groups were incubated under the same conditions for 24 h at 37°C in the anaerobic chamber. Samples were collected at 0, 6, 12, and 24 h, respectively, and collected samples were extracted (using methods described above) and were stored at −80°C until further analysis. The experiments were performed in triplicate.

#### 2.4.3 Non-targeted qualitative metabolomics analysis

Non-targeted qualitative metabolomics analysis was performed on an ultra-performance liquid chromatography to quadrupole time-of-flight (UPLC- Q-TOF) system (SCIEX Exion LC coupled to a SCIEX Triple TOF 6600+) along with an ACQUITY UPLC HSS T3 C18 (2.1 mm × 100 mm, 1.8 μm, Waters, Co., Milford, United States). The conditions of UPLC, including carrier gas, autosampler compartment temperature, column compartment temperature, mobile phase A, mobile phase B, injection volume, and the flow rate, were the same as described above. The gradient elution was programmed as follows: 0.0–1.5 min, 5.0% B; 1.5–15.0 min, 5.0%–100.0% B; 15.0–18.0 min, 100.0%–100.0% B; 18.0–19.0 min, 100.0%–5.0% B; 19.0–20.0 min, 5.0% B. The parameters of the electrospray ionization (ESI) source were set as follows: curtain gas, 30 psi; ion source gas 1, 55 psi; ion source gas 2, 55 psi; source temperature, 550°C; ion spray voltage, 5,500 V in positive mode and −4,500 V in negative mode; declustering potential, 60 V in positive mode and −60 V in negative mode; collision energy, 20, 35, and 50 eV in positive mode, and −20, −35, and −50 eV in negative mode. Information-dependent acquisition together with dynamic background subtraction was applied in the time-of-flight mass spectrometry data acquisition to simultaneously collect the full scan spectrum and the product ion spectra of the most abundant ions, enhancing the robustness of MS/MS data for metabolite identification.

#### 2.4.4 UPLC- Q-TOF data analysis

Raw data were acquired by Analyst 1.7.1 software (SCIEX, Foster City, CA, United States) and checked/viewed with PeakView 2.2 software (SCIEX, Foster City, CA, United States). Metabolites search and prediction were performed by MetabolitePilot™ 2.0.4 software using default settings (SCIEX, Foster City, CA, United States).

#### 2.4.5 Metabolite verification

Predicted metabolites of interest were selected for further verification by comparing their MS profiles (fragments and retention time) to reference standards solutions of S-acetylthiorphan and thiorphan (purchased from Sigma-Aldrich, St. Louis, MO). Standard solutions of 10 mg/mL (10 µL) were diluted in 10 mL of sterile ML medium and were subjected to the same procedures of sample extraction and non-targeted qualitative metabolomics analysis described above. The experiments were performed in triplicate.

### 2.5 Racecadotril-metabolizing capacity of LCZ in an *in vitro* simulated human digestion system

An advanced near-real dynamic *in vitro* human gastrointestinal (GI) digestion system (DHS-IV; Xiao Dong Pro-health Instrumentation Co., Ltd., Suzhou, China) was used to study the racecadotril-metabolizing capacity of LCZ.

The DHS-IV system comprised mainly the esophagus model, human stomach model, and human intestine model, and a lot of rolling-extrusion devices (Supplementary Figures S1A–C). These were silicone models created by 3D-printing technology and had similar dimensions, morphology, and anatomy to an actual human GI tract. The human digestive juices and GI kinetic parameters were prepared and set to simulate the human digestion process.

Three types of simulated digestive juices (simulated saliva fluid, gastric fluid, and intestinal fluid) were used to simulate the chemical environments in the respective models, which were prepared according to a previous study (Huang et al., 2020). Simulated saliva fluid (final pH adjusted to 7) was composed of human salivary α-amylase (75.0 U/mL), MgCl_2_(H_2_O)_6_ (0.15 mmol/L), KH_2_PO_4_ (3.7 mmol/L), NaHCO_3_ (13.6 mmol/L), KCl (15.1 mmol/L), and (NH_4_)_2_CO_3_ (0.06 mmol/L). Simulated gastric fluid (final pH adjusted to 1.6 with HCl) was composed of pepsin (250.0 U/mL), KCl (6.9 mmol/L), KH_2_PO_4_ (0.9 mmol/L), NaHCO_3_ (25.0 mmol/L), NaCl (47.2 mmol/L), MgCl_2_(H_2_O)_6_ (0.1 mmol/L), and (NH_4_)_2_CO_3_ (0.5 mmol/L). Simulated intestinal fluid (final pH adjusted to 7) was composed of pancreatin (200 U/mL), bile salts (8.17 g/L), KCl (6.8 mmol/L), KH_2_PO_4_ (0.8 mmol/L), NaHCO_3_ (85 mmol/L), and NaCl (38.4 mmol/L). The DHS-IV system was maintained at 37°C, and all components were sterilized before use.

Racecadotril (40 μL, 1 mg/mL) was added to 40 mL ML medium with or without pre-inoculated with LCZ (2 × 10^7^ CFU/mL). The cultures were mixed with 5 mL of simulated saliva fluid and then shaken in a water bath at 55 rpm, 37°C for 20 s to simulate the chewing action. The “chewed” oral digestive fluid mixtures were then transferred to the stomach system (pre-loaded with 10 mL of simulated gastric fluid to simulate the fasting state) through the esophagus model. During sample loading, simulated gastric fluid was continuously delivered to the stomach model by a syringe pump at a controlled rate of 1.75 mL/min for 60 min. Meanwhile, the pneumatically-controlled driving device was immediately activated, pressing the silicone stomach model at a rate of three compressions per minute by rolling extrusion plates. The pylorus sieving valve was simultaneously activated to squeeze the pylorus model at a constant speed of 8 mm/s, adjusting the opening size of the pylorus model in the range of 0–4.5 mm. The gastric digestive fluid passing through the pylorus model next entered the intestinal digestion system. In this process, simulated intestinal fluid was continuously injected in the intestinal digestion system at a controlled rate of 1.0 mL/min for 60 min. The intestinal model was compressed with six sets of rolling extrusion plates at a rate of 36 extrusions per minute. All parameters mentioned above were set according to previous studies (Peng et al., 2021; Wang et al., 2022) to mimic the human digestive process. Four milliliters of samples were collected at the end of the intestinal model at 6, 12, and 24 h. The experiment was performed in triplicate.

The total viable counts of LCZ were determined from serially diluted samples by the pour plate technique on ML agar. The inoculated ML agar plates were incubated anaerobically at 37°C for 48 h before counting the number of bacterial colonies. The survival rate of LCZ at different time points was calculated according to a previous study (Peng et al., 2020). The concentration of racecadotril, S-acetylthiorphan, and thiorphan was determined by the targeted quantitative metabolomics analysis described above.

### 2.6 *Ex vivo* degradation of racecadotril by LCZ as part of the personalized microbiome

#### 2.6.1 Fecal samples collection

The fecal samples were voluntarily provided by 26 healthy subjects recruited from the Key Laboratory of Dairy Biotechnology and Engineering, Inner Mongolia Agricultural University. The included subjects did not have diabetes, obvious GI, oral, or skin infections or diseases, malignancies, or a history of antibiotic use 3 months prior to or during sample collection. This study complied with the Chinese regulations regarding observational clinical studies and was examined and permitted by the Ethics Committee of the Affiliated Hospital of Inner Mongolia Medical University (No. KY2020006).

The fecal sample of each subject represented one personalized human gut microbiome. Fecal sample processing and *ex vivo* culture were performed according to the protocol reported by Javdan et al. (2020). Briefly, fresh human fecal samples were collected and transferred to an anaerobic chamber within 15 min of defecation. To make the frozen fecal glycerol stocks, fecal samples (1 g per sample) were re-suspended in 15 mL of sterile phosphate buffer supplemented with 0.1% L-cysteine, allowed to stand for 5 min, mixed evenly with an equal volume of 40% glycerol, and aliquoted (1 mL) in sterile cryogenic vials for storage at −80°C until use.

#### 2.6.2 16S rRNA gene amplicon sequencing analysis of personalized microbiome

The personalized microbiota of each subject was determined by sequencing the 16S rRNA amplicons generated from metagenomic DNA extracted from subjects’ fecal microbiota or the *ex vivo* fecal culture. To prepare for *ex vivo* fecal culture, 200 µL from each donor glycerol stock was inoculated into 20 mL of BG medium and cultured anaerobically at 37°C. The BG medium comprised the liquid BB medium and liquid mGAM medium in a ratio of 7:3. After 24 h, cultures were centrifuged at 13,000 g, 4°C for 10 min. The pellets and the corresponding fecal samples were used for DNA extraction with the PowerSoil DNA Isolation kit (QIAGEN, United States). The concentration and purity of the extracted DNA were monitored on 1% agarose gels. The V4 region of 16S rRNA was amplified using a specific primer pair, 515F and 806R (Walters et al., 2016). Illumina sequencing libraries were prepared using the TruSeq^®^ DNA PCR-Free Sample Preparation Kit (Illumina, United States). The library quality was assessed with a Qubit@ 2.0 Fluorometer (Thermo Scientific) and an Agilent Bioanalyzer 2100 system. The libraries were sequenced on an Illumina NovaSeq platform, and a dataset of 2 bp × 250 bp of an average depth of ∼50,000 reads was generated.

Raw sequencing reads were de-multiplexed based on sample barcodes, and the barcode and primer sequences were trimmed. Then, unmerged paired-end sequences were filtered and analyzed using QIIME 2 Core 2022.2 distribution (Bolyen et al., 2019). Taxonomy was assigned to the resulting amplicon sequencing variants with a naive Bayes classifier trained on the Greengenes database. Downstream analyses were performed in R (version 4.1.2) with the ggplot2 package.

#### 2.6.3 Personalized effect of LCZ on racecadotril degradation

The effect of exogenous addition of LCZ on racecadotril degradation by personalized microbiome was investigated. An aliquot of 200 µL of the glycerol stock was inoculated into 20 mL of BG medium for anaerobic cultivation at 37°C. After 24 h, 20 μL of racecadotril (1 mg/mL) was added to the culture together with or without 5 × 10^6^ CFU/mL of LCZ. In the control culture without LCZ inoculation, equal volume of BG medium was added instead. Additionally, there was also a drug-medium control (contained only racecadotril without any bacteria). Both the inoculated cultures and the controls were anaerobically cultured for another 24 h at 37°C. The concentration of the undegraded portion of racecadotril in the culture was determined by the targeted quantitative metabolomics analysis described above. The experiment was performed in triplicate.

## 3 Results

### 3.1 Five probiotic strains showed a variable drug-metabolizing capacity

**FIGURE 1:**
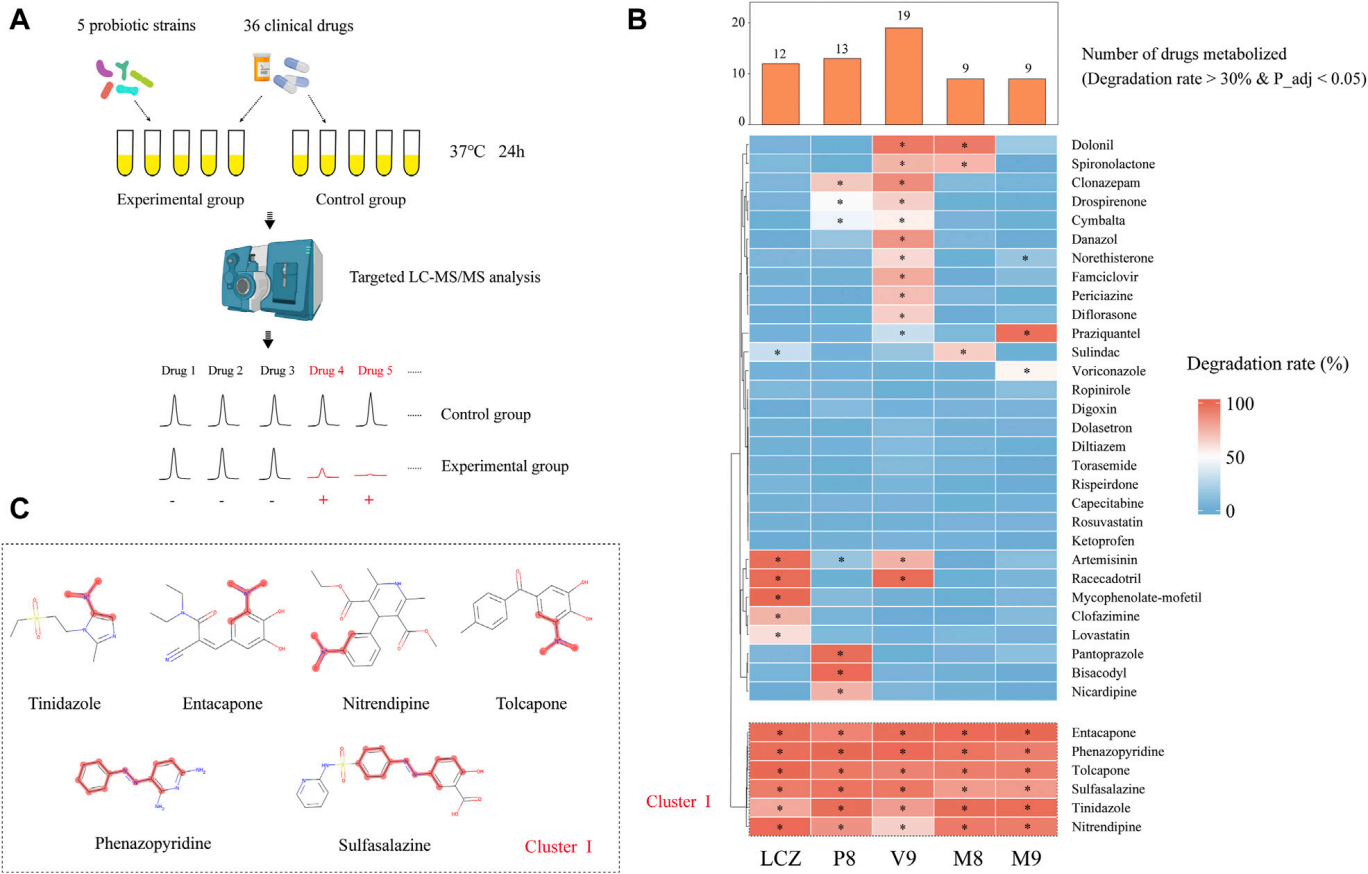
Drug-metabolizing activities of probiotics. **(A)** Schematic illustration of the assay. **(B)** Heat map showing the capacity of five probiotic strains (*Lacticaseibacillus casei* Zhang, LCZ; *Lactiplantibacillus plantarum* P8, P8; *Bifidobacterium animalis* subsp., *lactis* V9, V9; *B. animalis* subsp. *lactis* Probio-M8, M8; *Lacticaseibacillus rhamnosus* Probio-M9, M9) in metabolizing 36 drugs. Hierarchical clustering analysis was performed based on degradation rate, and Cluster I comprised drugs that could be metabolized by all five probiotic strains. The bar chart shows the number of drugs metabolized by each probiotic strain. * adjusted *p* < 0.05, Wilcoxon test. **(C)** Chemical structure of the six cluster I drugs. The common substructures of these drugs are shown in red, i.e., a nitro group in entacapone, tolcapone, tinidazole, and nitrendipine; and an azo group in phenazopyridine and sulfasalazine.

First, the capacity of five probiotic strains in metabolizing 36 commonly used clinical oral drugs was tested *in vitro* by incubating each drug with/without inoculating with each probiotic strain, and the drops in the drug concentrations after 24-h incubation with each probiotic strain compared with culture without bacterial inoculation were measured by targeted UPLC-QqQ-MS/MS (total number of tested probiotic-drug interactions, *n* = 36 drugs x 5 probiotic strains; 3 replicates per test; a total of 1,080 samples were analyzed, including the negative control; Figure 1A). These 36 drugs were chosen to be tested in this study because they are commonly used, and they have been reported to be metabolized by human gut bacteria (Zimmermann et al., 2019a). Supplementary Table S1 shows the optimized multiple reaction monitoring transitions, retention time, standard curve equation, and correlation coefficient (*R* ^2^) of each analyte. Good linearity was found in all drugs and standards with *R* ^2^ greater than 0.9900, indicating a good quantitative accuracy.

After the 24-h incubation with probiotic, a significant drug concentration drop (>30% degradation, adjusted *p* < 0.05; Figure 1B) was observed in 75% (27/36) of the assayed drugs. The V9 strain was able to metabolize the highest number (19/36) of drugs, followed by P8 (13 drugs), LCZ (12 drugs), M8 (9 drugs), and M9 (9 drugs). Notably, the profile of the drug-metabolizing capacity varied among the five tested probiotic strains. Three (mycophenolate-mofetil, clofazimine, and lovastatin), three (pantoprazole, bisacodyl, and nicardipine), four (danazol, famciclovir, periciazine, diflorasone), and one (voriconazole) drug could only be metabolized by LCZ, P8, V9, and M9, respectively, suggesting strain specificity in the drug-metabolizing capacity.

Notably, clustering based on the probiotic-metabolizing capacity identified six drugs (namely entacapone, phenazopyridine, tolcapone, sulfasalazine, tinidazole, and nitrendipine) that were metabolized by all five probiotic strains. Interestingly, these six drugs were found to share common substructures (a nitro group in entacapone, tolcapone, tinidazole, and nitrendipine; an azo group in phenazopyridine and sulfasalazine; Figure 1C), suggesting that these two functional groups could be the potential targets for the metabolic modifications by probiotics.

### 3.2 LCZ metabolized racecadotril into S-acetylthiorphan and thiorphan in monoculture

**FIGURE 2:**
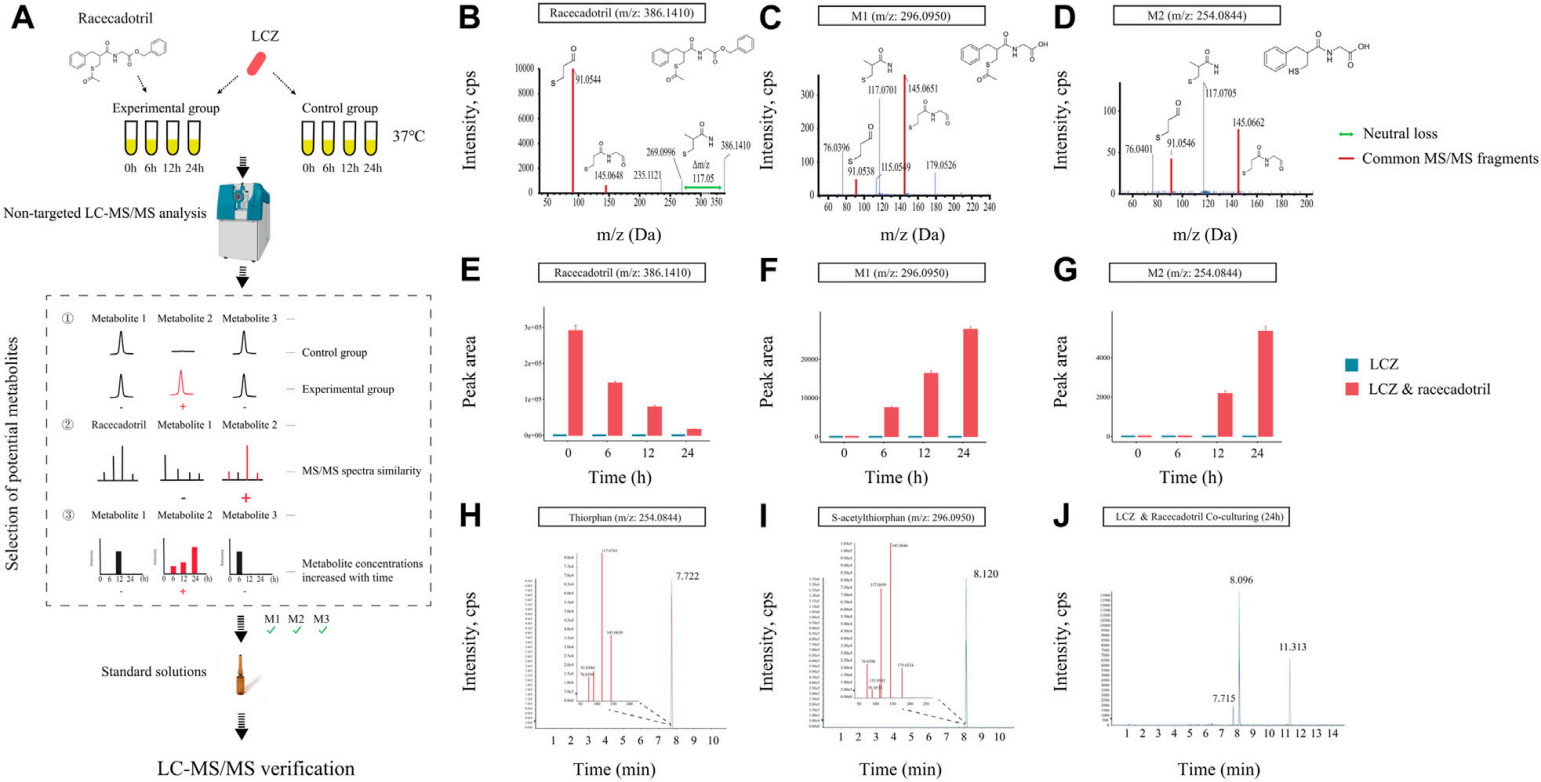
Identification of drug metabolites produced by *L. casei* Zhang (LCZ). **(A)** Schematic illustration of the assay. **(B–D)** Tandem mass (MS2) spectra of racecadotril and its metabolites, M1 and M2 (m/z = 296.0950, 254.0844, respectively). **(E–G)** The bar charts show the peak area of racecadotril and its candidate metabolites (M1 and M2) at different time points. Error bars represent SD. **(H,I)** Extracted ion chromatograms (XIC) of thiorphan and s-acetylthiorphan from authentic chemical standards. The insets show the enlarged peak clusters of their MS2 spectra. **(J)** The XIC of samples extracted from culturing LCZ in the presence of racecadotril, and the peaks corresponded to M2 (m/z: 254.0844), M1 (m/z: 296.0950), and racecadotril, respectively.

Microbial transformation can activate or inactivate drugs, or produce toxic compounds which can induce serious side effects on the host. Thus, it is important to identify probiotic-produced drug metabolites and characterize the biotransformation in each probiotic-drug interaction. A non-targeted LC-MS/MS-based assay was developed to identify drug metabolites (Figure 2A). A previous study reported that *L. casei* could improve diarrhea in children (Lai et al., 2019), and here we found that LCZ could metabolize racecadotril (Figure 1B) - a drug for treating diarrhea. So, the probiotic-drug interaction between LCZ and racecadotril was used to establish this protocol, which would provide valuable data for future use of probiotic-drug co-treatment.

First, the *in vitro* growth response of LCZ was evaluated. No significant difference was observed in the growth of LCZ regardless of the presence of racecadotril in the culture medium, suggesting that this drug did not influence the growth of LCZ in culture (Supplementary Figure S2). Based on the growth curves, it took approximately 6 and 12 h for LCZ to reach the logarithmic and stationary phases, respectively, and the bacterial cultures remained at the stationary phase until at least 24 h. Therefore, samples were collected at 0, 6, 12, and 24 h to monitor changes in the concentration of racecadotril and metabolite production by metabolomics analyses.

The concentration of racecadotril in the ML medium was monitored by targeted qualitative metabolomics analysis for auto-degradation. No significant change was observed in the concentration of racecadotril after 6-, 12-, and 24-h of incubation at 37°C compared with 0-h (Supplementary Figure S3), suggesting that racecadotril did not auto-degrade.

Meanwhile, changes in the concentration of racecadotril and the formation of its metabolites at these time points were monitored by non-targeted qualitative metabolomics analysis. The retention time of all samples showed good coincidence on the overlay TICs in both positive and negative modes (Supplementary Figures S4A, B), indicating good stability of the LC-MS system. Racecadotril was detected only in the positive but not the negative mode by searching its theoretical mass to charge ratio (m/z; Supplementary Figures S4C, D). Thus, the data acquired in positive mode was used for further metabolite searching through MetabolitePilot™ software. MetabolitePilot™ is a powerful software package that allows comparative analysis between two mass spectra for extracting compounds that appear only in samples but not controls, and it only extracts compounds that have common MS/MS fragments or neutral loss compared with the target compound (Supplementary Figure S5). In our analysis, a compound was deemed as a drug metabolite when it: 1) was only observed in the LCZ-racecadotril culture; 2) had common MS/MS fragments or neutral loss with racecadotril; 3) could be detected in three independent experiments and increased as the incubation prolonged.

At 0 h, only racecadotril but no other metabolites were detected in the LCZ-racecadotril culture (Supplementary Figure S6A). At 6 h, a new compound (referred to as M1; m/z = 296.0940, retention time = 8.09; Supplementary Figure S6B) was detected, and its peak size increased further at 12 h (Supplementary Figure S6C). At 24 h, in addition to M1, another new compound emerged (M2; m/z = 254.0644, retention time = 7.71 min; Supplementary Figure S6D). These two compounds had two MS/MS fragments (i.e., 145.0645 m/z and 91.0546 m/z) common to racecadotril, and racecadotril had one neutral loss [Δ (386.1426–269.0940) m/z], which is the same as the fragment seen in the mass spectra of M1 and M2 (corresponding to 117.0696 m/z). Their possible chemical structures were predicted by the MetabolitePilot™ software (Figures 2B–D; Supplementary Figure S6). The levels of these two compounds increased as the incubation prolonged, accompanied by corresponding decreases in racecadotril (Figures 2E–G; Supplementary Figures S7A, B). The M2 peak was not picked up by MetabolitePilot™ at 12 h due to its low signal intensity (intensity <1,000; Supplementary Figure S7C) and lack of MS/MS fragments data.

Based on the mass spectra features and chemical structures, M1 and M2 were predicted to be S-acetylthiorphan and thiorphan, respectively. Thus, corresponding standard solutions were purchased from Sigma-Aldrich for UPLC-Q-TOF analysis under the same conditions as the sample runs, confirming that S-acetylthiorphan and thiorphan had identical mass, retention time, and MS/MS fragments as M1 and M2, respectively (Figures 2H–J; Supplementary Figures S8A, B). These results confirmed that S-acetylthiorphan and thiorphan were the drug metabolites produced by LCZ from racecadotril.

### 3.3 LCZ metabolized racecadotril in a dynamic simulated digestion system

Probiotics are not the native inhabitants of the human GI tract. Thus, it is essential to study the capacity of probiotics in drug metabolism together with their viability during the GI transit. A near-real dynamic *in vitro* human GI digestion system (DHS-IV) was applied to further validate the ability of LCZ in metabolizing racecadotril meanwhile monitoring changes in the bacterial viability. Samples were collected at the end of the intestinal model at 6, 12, and 24 h to determine the bacterial viability by plate counts. Our results showed that the viable counts of LCZ dropped with time in the *in vitro* digestion system (59.68%, 41.07%, and 25.21% at 6, 12, and 24 h, respectively; Supplementary Table S2).

**FIGURE 3:**
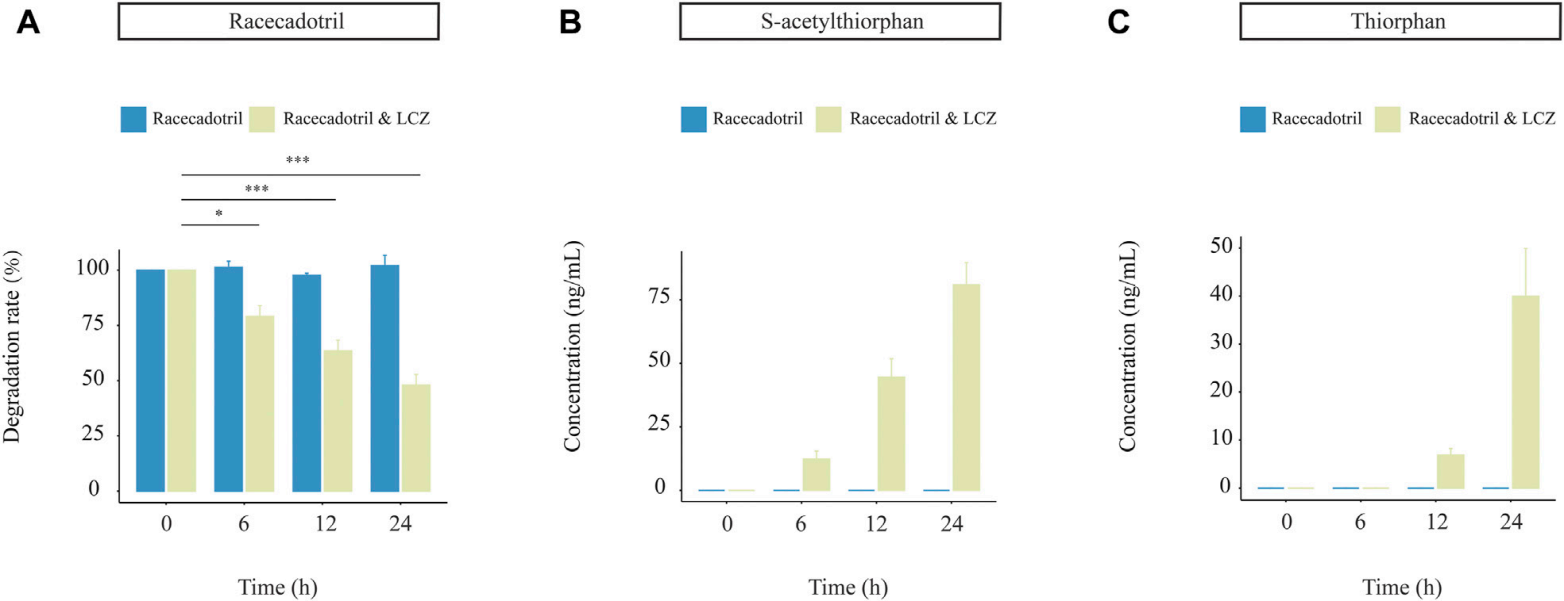
Drug-metabolizing activities of *L. casei* Zhang (LCZ) in a near-real human digestion system (DHS-IV). **(A)** racecadotril and **(B,C)** changes in concentrations of s-acetylthiorphan and thiorphan at different time points after digestion in the *in vitro* human simulated digestion system in the presence or absence of LCZ. The drug and metabolites were detected by targeted metabolomics. Error bars represent SD. ***p < 0.001, *p < 0.05, Wilcoxon test.

Meanwhile, the concentration of racecadotril in the *in vitro* simulated human digestion system dropped significantly in the presence of LCZ (*p* < 0.05; average degradation rate = 77.23%, 61.54%, and 49.56% compared with the baseline; Figure 3A), and such drop was not observed in the negative control without adding LCZ. Similarly, the decrease in racecadotril was accompanied by the increases in the concentrations of s-acetylthiorphan (detected at 6, 12, and 24 h; Figure 3B) and thiorphan (only detected at 12 and 24 h; Figure 3C) in the presence of LCZ. Again, such changes were not observed in the negative control without LCZ.

### 3.4 LCZ exhibited personalized effects on racecadotril metabolism

**FIGURE 4:**
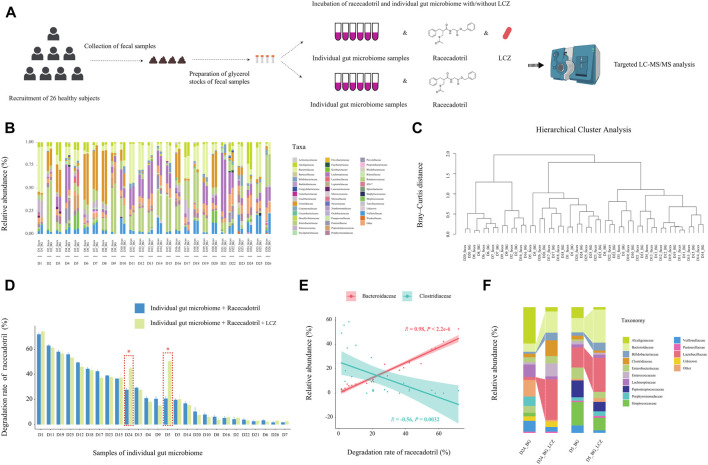
Effect of individuality of gut microbiome on *L. casei* Zhang (LCZ)-driven drug degradation. **(A)** Schematic illustration of the assay. **(B)** Family-level bacterial microbiota composition of fecal samples in comparison with their *ex vivo* fecal culture counterparts of 26 subjects (D1-26; the prefix sample code represents the subject number, and the suffix code represents type of sample, i.e., feces or *ex vivo* fecal culture in BG medium [BG]; BG medium comprised liquid BB medium and liquid mGAM medium in a ratio of 7:3). “Other” represents taxa that were below 1% of total sequences. **(C)** Hierarchical cluster analysis of microbiota profile of fecal samples and their *ex vivo* fecal culture counterparts. **(D)** The bar chart compares differences in racecadotril degradation rate in *ex vivo* fecal culture of each individual in the presence/absence of LCZ. The concentration of racecadotril was detected by targeted metabolomics. Error bars represent SD. * adjusted *p* < 0.05, Wilcoxon test. **(E)** Correlation (Pearson’s) between racecadotril degradation rate and *Bacteroidaceae*/*Clostridiaceae*. **(F)** Family-level bacterial composition of *ex vivo* fecal cultures of subjects D5 and D24 before and after incubation with LCZ for 24 h.

Our data so far suggested that LCZ had the capacity of metabolizing racecadotril in monoculture; however, whether its drug biotransformation capacity would be affected by the great complexity and compositional individuality of the human microbiome when present as part of the gut microbial community remained to be answered. Thus, the capacity of LCZ in metabolizing racecadotril was tested in an *ex vivo* fecal co-culture system (Javdan et al., 2020) of stool samples collected from 26 healthy human donors (Figure 4A).

First, the 16S rDNA amplicon profiles of the fecal microbiota and *ex vivo* fecal culture microbiota were compared to ensure that the *ex vivo* culture procedure would not cause significant bias in preferentially expanding only few taxa (Figure 4B). Our results showed that the family-level compositional profiles of the original fecal microbiota and *ex vivo* culture of each subject were largely similar in terms of bacterial relative abundance and diversity, which was also confirmed by unsupervised hierarchical cluster analysis, showing the shortest dissimilarity distance between the fecal microbiota and their *ex vivo* culture counterparts in most subjects, and they are joining together as a pair in the dendrogram (Figure 4C). These results suggested that the *ex vivo* culturing system supported the growth a wide variety of the intrinsic fecal microbiota and was representative of the original personalized fecal microbiome of each subject.

Next, the ability of the *ex vivo* fecal culture (with/without exogenous addition of LCZ) to metabolize racecadotril was investigated. Notably, around two-thirds (18/26; the range of degradation rate = 7.72%–71.83%, adjusted *p* = 0.0002 to 0.0286; Supplementary Figures S9; Figure 4D) of the samples (without the addition of LCZ) showed varying but significant degree of racecadotril degradation into S-acetylthiorphan and thiorphan, compared with the blank control; and, in most cases (24/26), the addition of LCZ did not exhibit significant differences in racecadotril degradation compared with the natural degradation rate (adjusted *p* > 0.05). Interestingly, the supplementation of LCZ in two *ex vivo* human fecal culture samples, D5 and D24, significantly enhanced racecadotril degradation (adjusted *p* < 0.05; Figure 4D).

To identify potential taxa that might be responsible for the racecadotril metabolism, correlation analysis was performed. The relative abundance of *Bacteroidaceae* was positively correlated with the degradation rate of racecadotril (*R* = 0.95, *p* < 2.2e-6; Figure 4E; Supplementary Figure S10), and the formation of thiorphan (*R* = 0.91, *p* = 1.5e-5) and S-acetylthiorphan (*R* = 0.86, *p* = 0.0013; Supplementary Figure S12A). In contrast, the relative abundance of *Clostridiaceae* was negatively correlated with the degradation rate of racecadotril (*R* = −0.56, *p* = 0.0032; Figure 4E; Supplementary Figure S10), and the formation of thiorphan (*R* = −0.50, *p* = 0.0095) and S-acetylthiorphan (*R* = −0.56, *p* = 0.0029; Supplementary Figure S12B).

On the other hand, to find out whether the 24-h exogenous addition of LCZ had any effect on the microbiota composition of the *ex vivo* cultures D5 and D24, metagenomic DNA from these incubated co-cultures was extracted for comparative V4-16S-rRNA-based microbiota analysis against its pre-incubation counterpart. After 24-h incubation with LCZ, the relative abundance of *Bifidobacteriaceae*, *Bacteroidaceae*, and *Lactobacillaceae* increased significantly only in D5 and D24 *ex vivo* cultures (Figure 4F, *p* < 0.05) but not other samples (Supplementary Figure S11, *p* > 0.05).

## 4 Discussion

Current applications of probiotics are not limited to food and cosmetic products, but are also increasingly used by clinicians to ameliorate symptoms and/or as adjuvant therapeutics in various diseases. Probiotics presented good clinical efficacies in GI dysbiosis, metabolic diseases, and even neurological disorders (Aponte et al., 2020). The therapeutic effect of probiotics is usually not as direct, fast, and effective as drugs; therefore, they are often used in combination with other drugs as therapeutic adjuvants rather than drug replacement. One of the proposed symptom-alleviating mechanisms of probiotics is their ability to restore a healthier gut microbiota from disease-associated dysbiotic states. Increasing evidence supports that the gut microbiota is involved in drug metabolism; thus, as a part of the regular gut microbiota after being ingested, probiotics are anticipated to take part in drug interaction as well. Few studies have yet addressed the drug metabolizing effects of ingested probiotics.

In this study, we first demonstrated that 75% (27/36) of commonly used oral drugs could be variably metabolized by five probiotic strains. Notably, the drug-metabolizing profile differed greatly between strains, suggesting that the drug-metabolizing activity of probiotics is strain-specific. Such variation, on the one hand, might reflect the different original niches of the tested strains [V9 isolated from a stool sample (Sun et al., 2010); M8 and M9 isolated from human breast milk (Zhang et al., 2020; Xu et al., 2021); LCZ and P8 isolated from traditional fermented dairy products (Ya et al., 2008; Wang et al., 2015)] and their environmental/metabolic adaptation. For example, a previous large-scale comparative genomic study of 455 *L. plantarum* genomes identified clear habitat-specific features; isolates obtained from fermented dairy products, animal and human gut/clinical specimens were found to contain multiple environment-specific genes (Li et al., 2022). In contrast, many early life-associated bifidobacteria isolated from breastfed infant gut, presumably acquired from mother’s milk, are functionally specialized in metabolizing human milk (Lawson et al., 2020). The V9 strain was originally isolated from a human stool sample. Provided the repetitive exposure of the human GI tract to a wide variety of drugs, it is not surprising that V9 could metabolize the most assayed drugs among the tested strains. On the other hand, the chemical structures of the drugs could be an important factor in determining whether they could be easily metabolized. Six of the assayed drugs were found to be metabolized by all five probiotic strains, and common to these drugs is the presence of a nitro or an azo group, rendering them degradable under anaerobic conditions, possibly by azoreductases and nitroreductases that are frequently present across lactobacilli, bifidobacteria, and other anaerobes (Marteau et al., 1990). Thus, the source of probiotics and the functional groups carried by the drugs are important information to guide the combined use of probiotics and drugs.

The metabolism of drugs by probiotics can alter their structure, changing their bioavailability, bioactivity, and toxicity, which will inevitably lead to concerns about therapeutic efficacy and safety. Therefore, we conducted an untargeted metabolomics analysis to identify probiotic-produced drug metabolites to gain insights into the biotransformation process. Two key differences set our approach apart from previous studies. First, instead of collecting samples at the end of the incubation, samples were collected at multiple time points to reduce false-positive. Second, previous studies always relied on searching across existing tandem mass spectral (MS2) databases, such as KEGG, HMDB, METLIN, mzCloud, and MassBank, for metabolite identification, which are collections of data from different platforms, such as QTOF, Orbitrap, and QqQ, with widely varied instrumental conditions, e.g., collision energy and column types. These variations greatly limit the accuracy of the identification. Thus, this study used MetabolitePilot™ software to identify the metabolites of interest, followed by experimental verification of the retention time, accurate precursor mass (MS1), and MS2 spectra with authentic chemical standards under the same conditions, which is the “gold” standard for metabolite identification (Vinaixa et al., 2016). By untargeted metabolomics analysis, it was confirmed that racecadotril was metabolized into S-acetylthiorphan and thiorphan by LCZ. S-acetylthiorphan and thiorphan are reported active metabolites of racecadotril (Spillantini et al., 1986; Eberlin et al., 2012), implicating that the metabolic transformation of LCZ can contribute to the bioactivity of racecadotril. It was reported that the liver can metabolize thiorphan into inactive metabolites such as sulfoxide of S-methylthiorphan, S-methyl thiorphan, 2-methane-sulfinyl methyl propionic acid, and 2-methyl sulfanyl methyl propionic acid *via* cytochromes P450 enzymes (Eberlin et al., 2012). Those inactive metabolites were not identified in this study. A possible reason for this is that P450 enzymes are generally not present in *Lactobacillaceae* (Padayachee et al., 2020).

Both the probiotics and oral drugs would pass through the harsh conditions in the GI tract, including challenges under low pH, exposure to multiple digestive enzymes and bile salts, and prolonged peristaltic churning and mixing actions in their journey through the mouth, stomach, and intestine. These physical and chemical factors may directly affect probiotic-drug interactions. So, a dynamic GI model which simulated both the biochemical and mechanical processes of human digestion was used to validate the ability of LCZ in metabolizing racecadotril. The bacterial survival rate in the digestate collected at the end of the human intestine model was around 25% after 24-h of simulated digestion, suggesting that LCZ could tolerate human digestive juices and mechanical churning for a sufficiently long time. The survival rate of LCZ observed in this model was lower than that reported in previously static simulated GI juice tolerance tests (including sequential testing of tolerances to GI juices and bile; and evaluation of bile salt hydrolase activity) (Wu et al., 2009), indicating that traditional probiotic screening methods could overestimate the survival of probiotics through the human GI tract. Particularly, the mechanical churning stress is not considered in conventional assaying methods. Consistently, S-acetylthiorphan and thiorphan were mainly detected in the digestate of the intestinal model in the presence of LCZ but not in the negative control without bacterial inoculation. These results together suggested that LCZ could reach the intestine alive and remain active in racecadotril degradation. Moreover, the observation of the gradual decrease in racecadotril accompanied by progressive increases in S-acetylthiorphan and thiorphan in both the monoculture assay and simulated human digestive system suggested that the biotransformation process is a continuous process occurring only in the presence of LCZ.

We then investigated the racecadotril degradation ability of LCZ in an *ex vivo* fecal co-culture system, which simulated the natural colonic environment where probiotics would indeed become part of the host gut microbiome, at least temporally. Notably, the *ex vivo* fecal cultures showed variable racecadotril degradation ability without exogenous addition of LCZ, suggesting that the gut microbiota can naturally metabolize racecadotril, though such capacity is personalized due to the highly individualized gut microbiota composition. Differences in the host gut microbiome have been shown to lead to inter-individual phenotypic variations in digestive capacity and pharmacokinetic/pharmacodynamic responses (Zhu et al., 2015). We further found that the degradation rate of racecadotril had a strong positive correlation with *Bacteroidaceae* while negatively correlated with *Clostridiaceae*, highly suggestive of an active role of these two bacterial families in racecadotril degradation. Our result is consistent with Zimmermann et al. (2019a)), reporting that racecadotril could be fully metabolized by human gut isolates belonging to the phylum *Bacteroidetes*. Interesting, the exogenous addition of LCZ to the *ex vivo* fecal culture with racecadotril did not significantly increase the drug degradation rate in most cases, except for the fecal cultures of donors 5 and 24, suggesting that the biotransformation of racecadotril by an individual strain in a microbiota community is a lot more complicated than in monoculture. It is possible that the growth and gene expression of LCZ were somehow suppressed by the fecal microbes present in the co-culture environment in most cases. However, tracking strain-level microbial dynamics and gene expression changes remain technically difficult, which need to be addressed in future studies. Our results also did not rule out the chance that the drug metabolism could be contributed partly by the endogenous microbiota of the fecal culture. The current study was only able to show that the increase in racecadotril degradation in the LCZ-fecal co-cultures of donors 5 and 24 was accompanied by a significant expansion of the families *Bacteroidaceae*, *Bifidobacteriaceae*, and *Lactobacillaceae*. The taxonomic resolution of our 16S rRNA analysis was limited to the family level, but presumably at least part of the *Lactobacillaceae* enrichment was a direct effect of LCZ inoculation and growth. Our previous work found strong positive correlations between LCZ and specific taxa, including *Bifidobacteriaceae*, *Lactobacillaceae,* and *Bacteroidaceae*, in human gut (Zhang et al., 2014). The positive correlations might suggest symbiotic and even mutually stimulating relationships among these taxa when living under the provided environmental conditions. Thus, a microbiota community with a higher proportion of *Lactobacillaceae* (relative abundance >1%; Supplementary Figure S11) may benefit the growth of LCZ, which will in turn stimulate the growth of *Bifidobacteriaceae* and *Bacteroidaceae*. On the other hand, *Clostridiaceae* showed a strong negative correlation with the racecadotril degradation rate, suggesting its potential role in inhibiting drug degradation reaction. The relative abundance of *Clostridiaceae* in the LCZ-ex vivo fecal co-cultures of donors 5 and 25 showed opposite trends after the 24-h incubation with racecadotril (expanded in the co-culture of donor 24, increased from 3.25% to 9.43%; diminished in the co-culture of donor 5, decreased from 2.16% to 1.37%; Figure 4F). The contradictory result could again be due to the limited taxonomic resolution of our 16S rRNA analysis. *Clostridiaceae* is a highly diverse family, including a number of genera that contribute to different nutrient digestibility (Bermingham et al., 2017). It is possible that the observed responses in *Clostridiaceae* only represented some of the genera within this family. After all, these observations together suggested that the drug-metabolizing action of LCZ is greatly dependent on its interactions with the environmental microbiota.

These results provide important insights: 1) previous *in vitro* studies showing drug metabolism of single bacterial strains should be carefully interpreted and needed to be reproduced in assay conditions taken into account of the complex interactions in the gut microbial community; 2) probiotic applications, particular in clinical scenarios, should not be treated as “one-size-fits-all” supplement but as “precision probiotics” for the best benefits of patients and consumers (Sakandar and Zhang, 2022).

**FIGURE 5:**
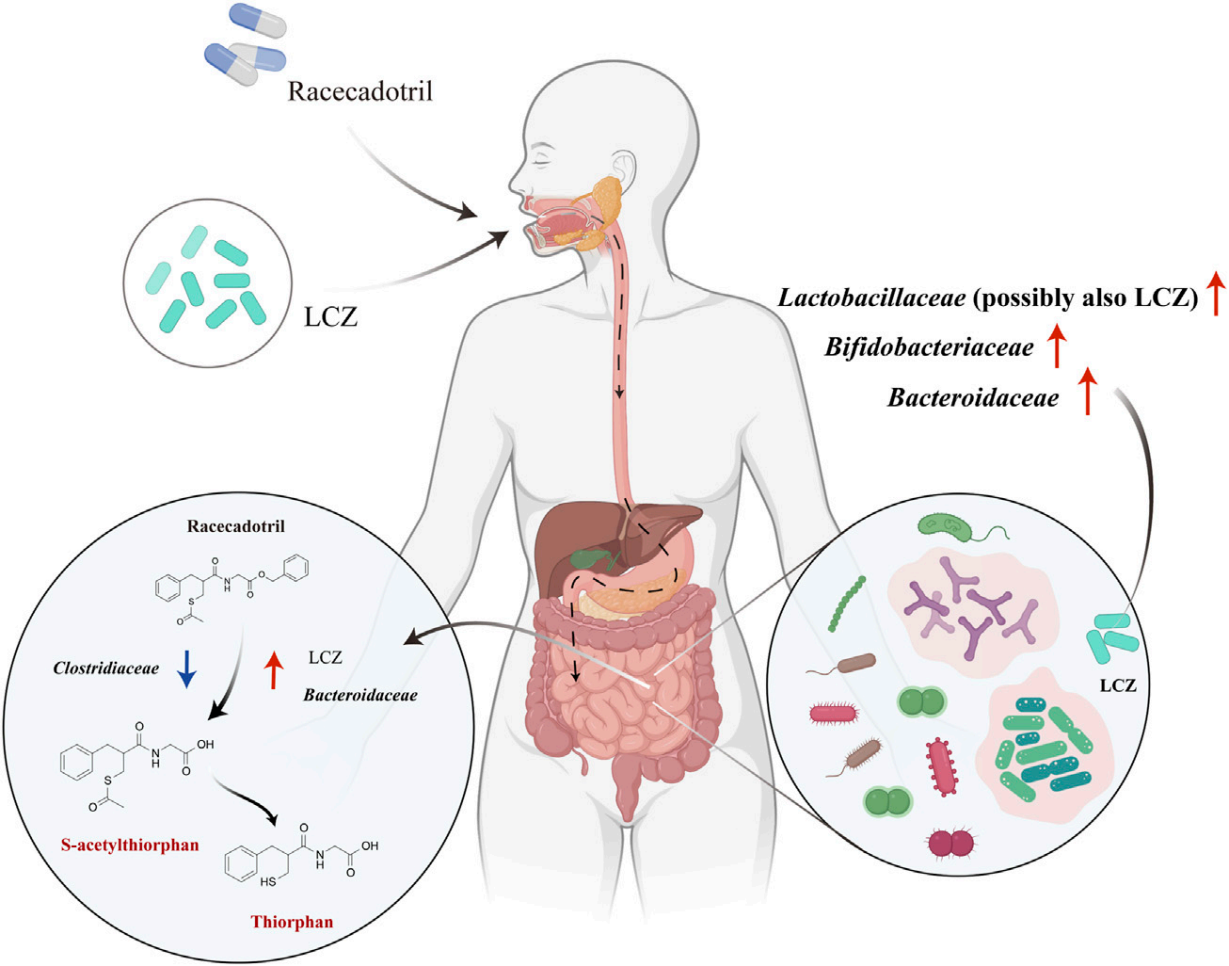
Proposed mechanisms of *in vivo* racecadotril degradation *via* direct metabolic action of *L. casei* Zhang (LCZ) and interactions between LCZ and host gut microbiome. Red arrows in the right circle indicate the effects of LCZ on the gut microbial communities. Arrows in the left circle indicate the effects of *Bacteroidaceae*/*Clostridiaceae/*LCZ on the metabolism of racecadotril (Red arrows indicate promotion, blue arrows indicate inhibition). Chemicals marked in red are metabolites of racecadotril verified by targeted metabolomics.

Based on our findings, we propose a possible *in vivo* mechanism of interactions between LCZ, racecadotril, and host gut microbiota (Figure 5). Viable LCZ cells transit through the harsh conditions in the stomach and the small intestine, gaining access to the colon. In the colon environment, the growth and metabolism of LCZ are favored, in the presence of some symbiotic and mutually supportive gut commensals, e.g., *Bifidobacteriaceae*, *Bacteroidaceae*, and particularly *Lactobacillaceae*. The expansion of these taxa, particularly *Bacteroidaceae* and possibly LCZ, enhances racecadotril metabolism into its active products, S-acetylthiorphan and thiorphan.

Although the current study design has already taken into account of multiple factors, such as probiotic specificity, different types of drugs, and the complex physiological environment of the human gastrointestinal tract, some important aspects in clinical drug therapy have still not been considered. For example, whether multiple administration of racecadotril (or other drugs, particularly those that are known to have a gut microbiota modulatory effect, e.g., proton pump inhibitors, metformin, selective serotonin reuptake inhibitors and laxatives) (Weersma et al., 2020) can change the capacity of LCZ and/or microbiota to metabolize the drug; and whether single or multiple probiotics administration could change the microbiota. Generally, combined probiotic formulations are considered more effective than single-strain probiotic products in preventing and managing diseases (Mathipa and Thantsha, 2015). These are relevant but complicated aspects that should be addressed in further studies. Finally, future experiments should target to increase the sample size and track microbial dynamics at a finer taxonomic resolution, ideally to a strain-level precision, which would further confirm the findings of this work and provide insights into microbiota-probiotic-drug interactions.

## 5 Conclusion

Nowadays, interest in using probiotics for preventing and treating multiple diseases is growing. Many probiotics are now used as adjuvants to drug therapy. This work provided a workflow for the first time to systematically study probiotic-drug interactions. By using the established framework, we demonstrated that probiotics could degrade commonly used drugs through their own metabolism and/or *via* modulating the gut microbiota in a host selective manner. Although the established workflow does not reveal the effect of probiotic intake on the bioavailability, bioactivity, and toxicity of drugs, it provides practical reference information for the combined use of probiotics and drugs, as well as valuable data for designing *in vivo* validation assays. We envisage that incorporating metabolomics, bionics, and especially culturomics would help elucidate inter-individual variabilities in drug responses and promote the transition to a precision probiotic use approach.

## Data Availability

The datasets presented in this study can be found in online repositories. The names of the repository/repositories and accession number(s) can be found in the article/Supplementary Material.
