# Supplementary material for: Integrating metabolomics, bionics, and culturomics to study probiotics-driven drug metabolism
Source: Front Pharmacol. 2023 Jan 26;14:1047863. doi: 10.3389/fphar.2023.1047863 (PMC9908756; doi:10.3389/fphar.2023.1047863)
Supplement: Supplementary file 3 [file DataSheet1.DOCX]

Supplementary Material


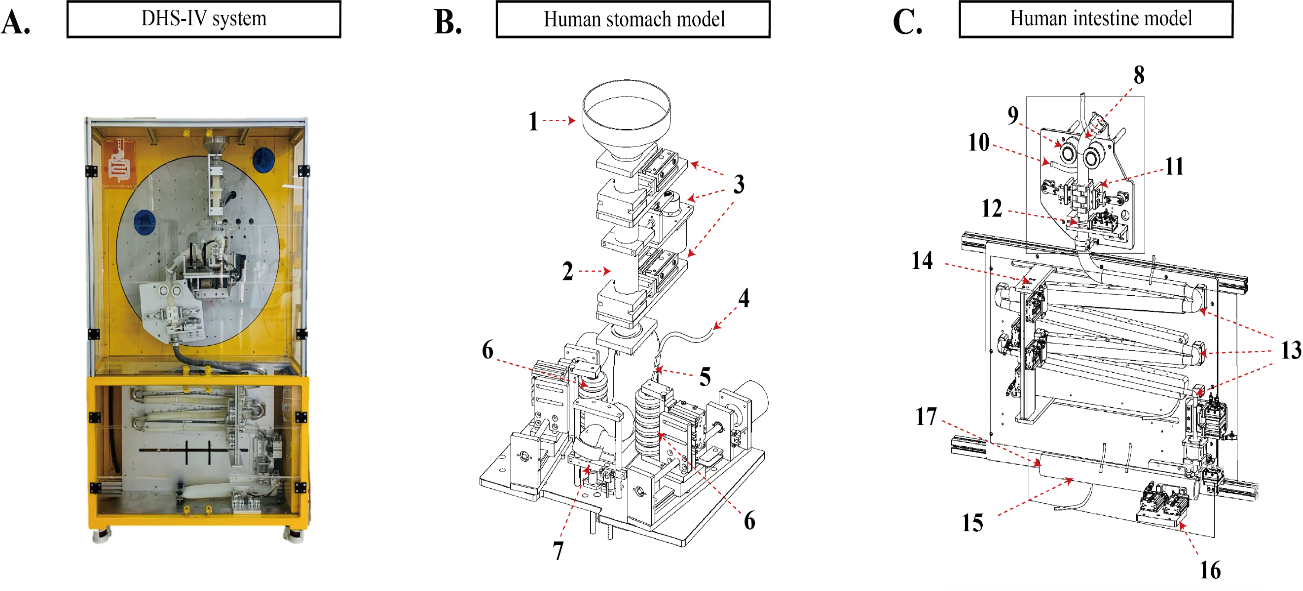


**Figure S1** The near-real human digestion system (DHS-IV). (A) The image of DHS-IV. (B, C) Schematic diagrams of the human stomach and intestine models of the DHS-IV. 1: Sample loading port; 2: Silicone esophagus model; 3: Esophagus vibrator; 4: Gastric secretion tube; 5: Silicone human stomach model; 6: Stomach rolling-extrusion device; 7: Pylorus valve; 8: Silicone human [duodenum](javascript:;) model; 9: [Duodenum](javascript:;) rolling-extrusion device; 10: [Intestinal](javascript:;) [juice](javascript:;) secretion tube; 11: [Duodenum](javascript:;) extrusion device; 12: [Duodenum](javascript:;) valve; 13: Silicone human intestine model; 14: Intestine rolling-extrusion device; 15: Silicone human colon model; 16: Colon rolling-extrusion device; 17: Location for sampling.


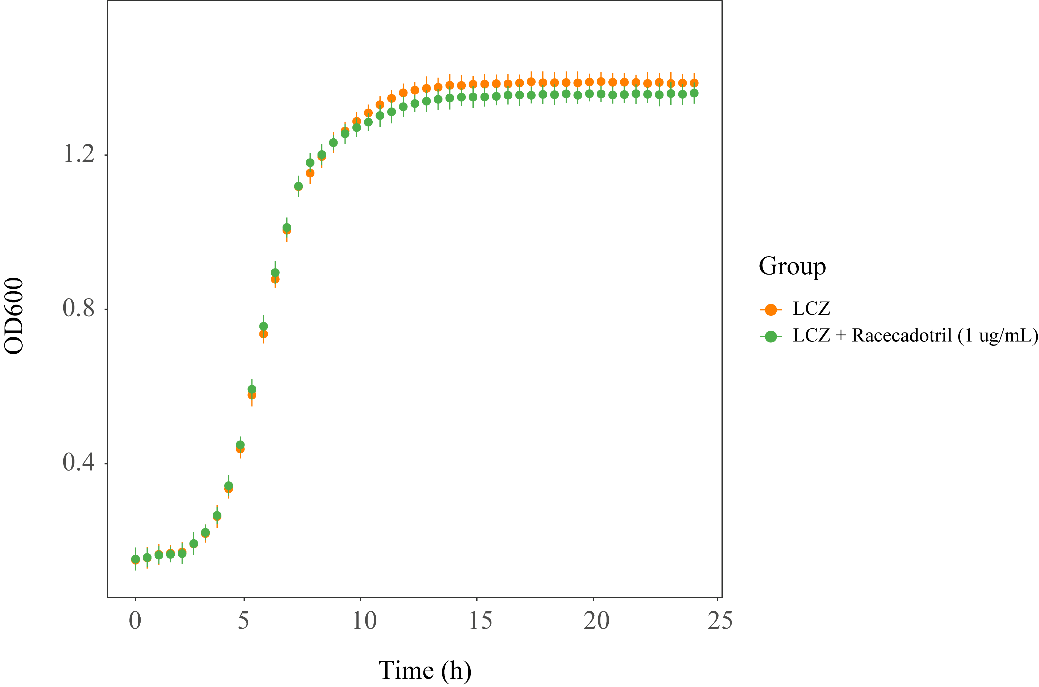


**Figure S2** Growth curves of *Lacticaseibacillus casei* Zhang (LCZ) with and without racecadotril. Error bars represent standard deviation.


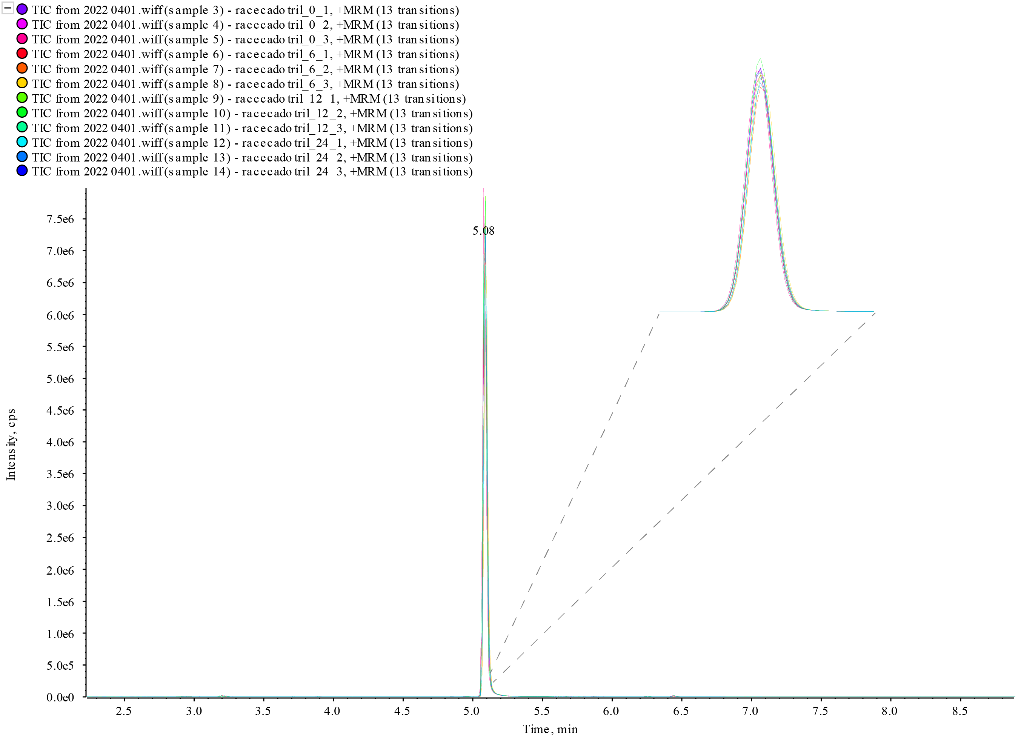


**Figure S3** The overlay total ion chromatogram (TIC) of racecadotril incubating at 37 ℃ for 0, 6, 12, and 24 hours. Data were acquired by targeted metabolomics through multiple reaction monitoring (MRM). The inset (zoomed in at the top-right corner) shows the peak cluster of racecadotril.


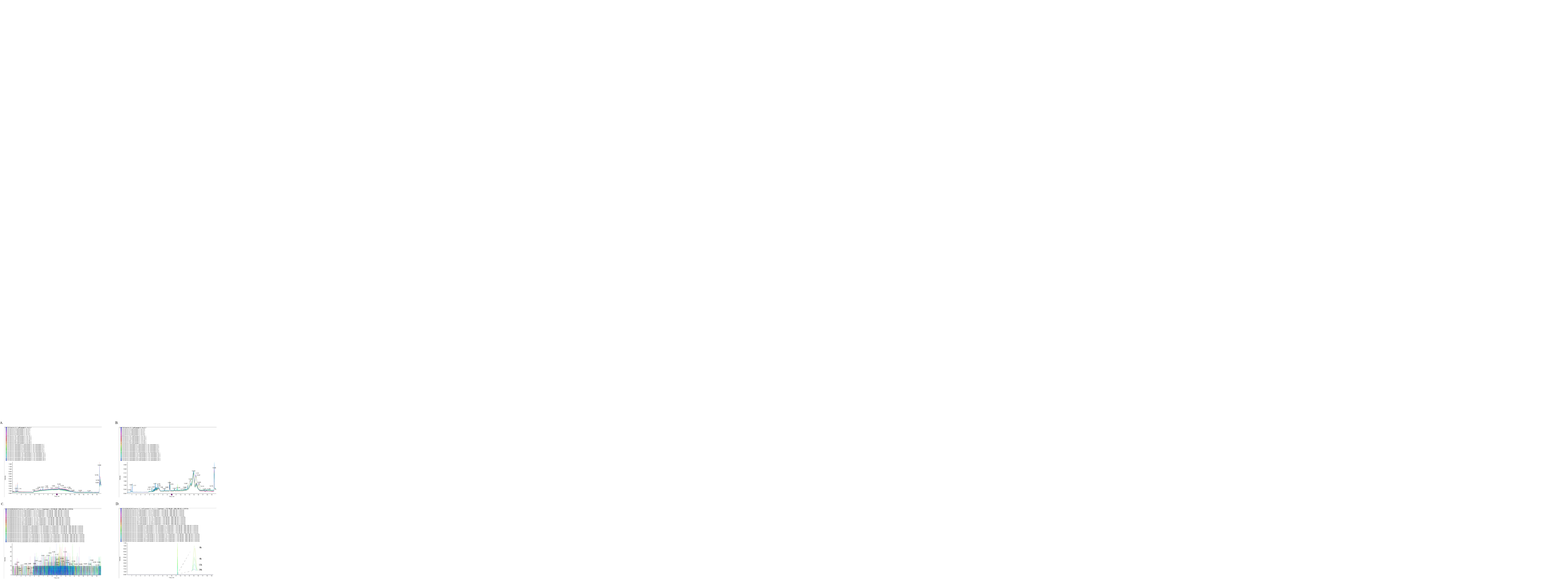


**Figure S4** Non-targeted metabolomics time course analysis of culture [inoculate](javascript:;)d with *Lacticaseibacillus casei* Zhang (LCZ) with or without adding racecadotril. The overlay of total ion chromatograms (TICs) of all samples in (A) negative and (B) positive modes; and the overlay of extracted ion chromatograms (XICs) of racecadotril from all samples in (C) negative and (D) positive modes. The inset in (D) shows the enlarged peak cluster at 11.32-minute retention time.


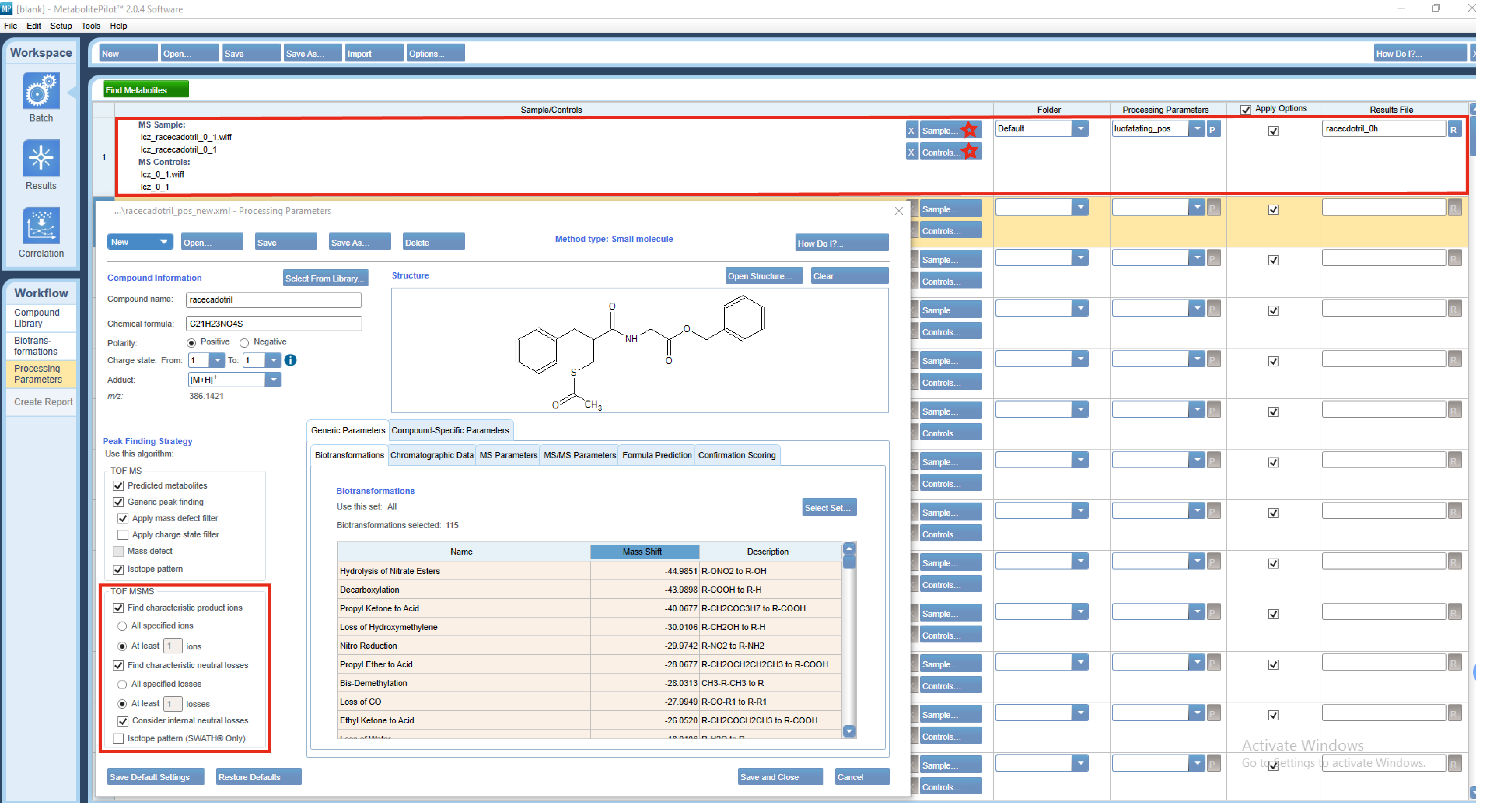


**Figure S5** The image of parameter settings of MetabolitePilot™ software.


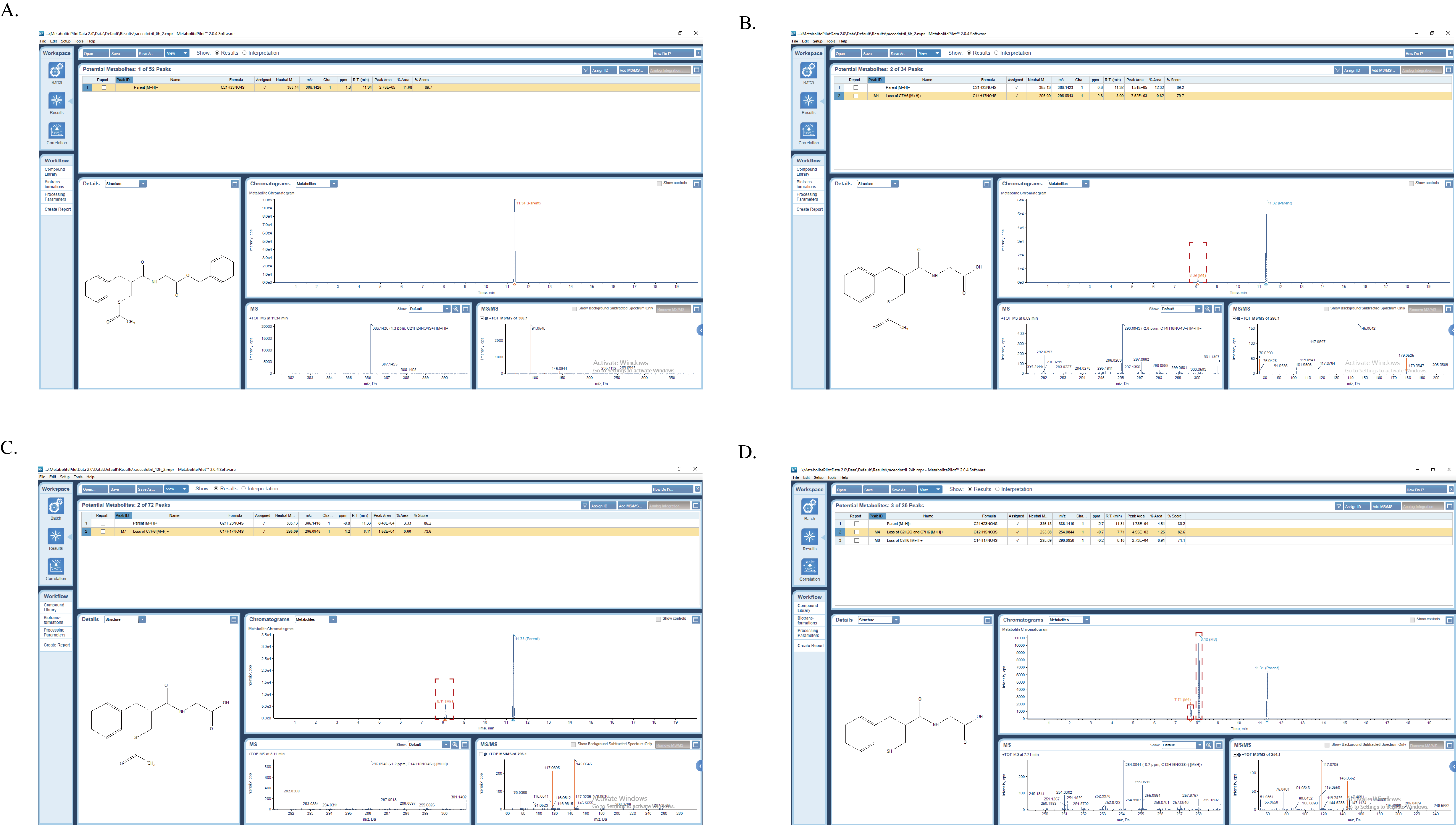


**Figure S6** Metabolite identification results obtained through MetabolitePilot™. Compounds were discovered at (A) 0 h, (B) 6 h, (C) 12 h, and (D) 24 h. The information of each compound, including accurate precursor mass (MS1), retention time, tandem mass (MS2) spectra, peak area, and predicted structure, is shown.


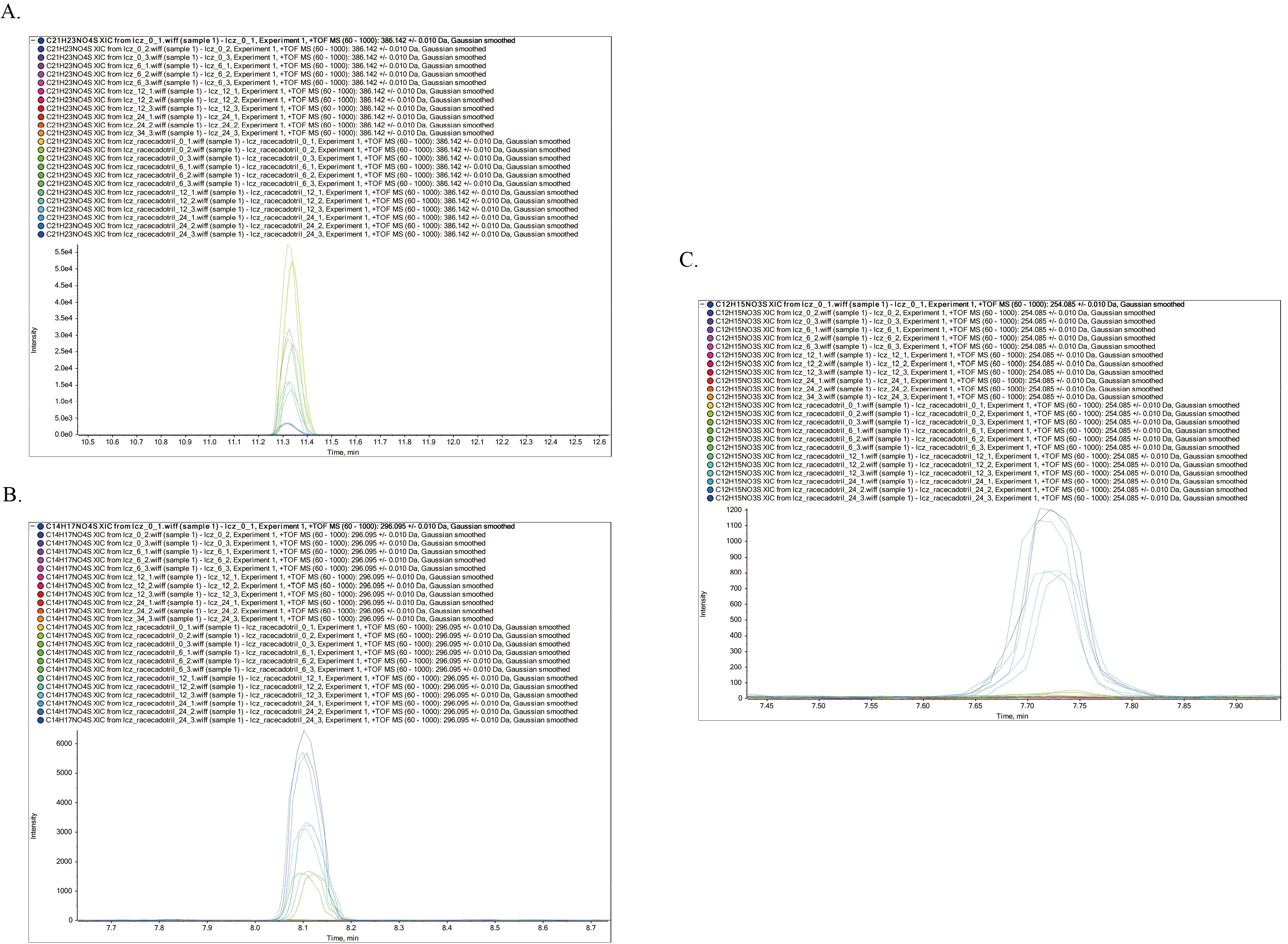


**Figure S7** Signal intensity of racecadotril and its [candidate](javascript:;) metabolites acquired by non-targeted metabolomics analysis. The overlay of extracted ion chromatogram (XIC) of (A) racecadotril, (B) candidate metabolite M1 (m/z: 296.0950), and (C) [candidate](javascript:;) metabolite M2 (m/z: 254.085) from all samples.


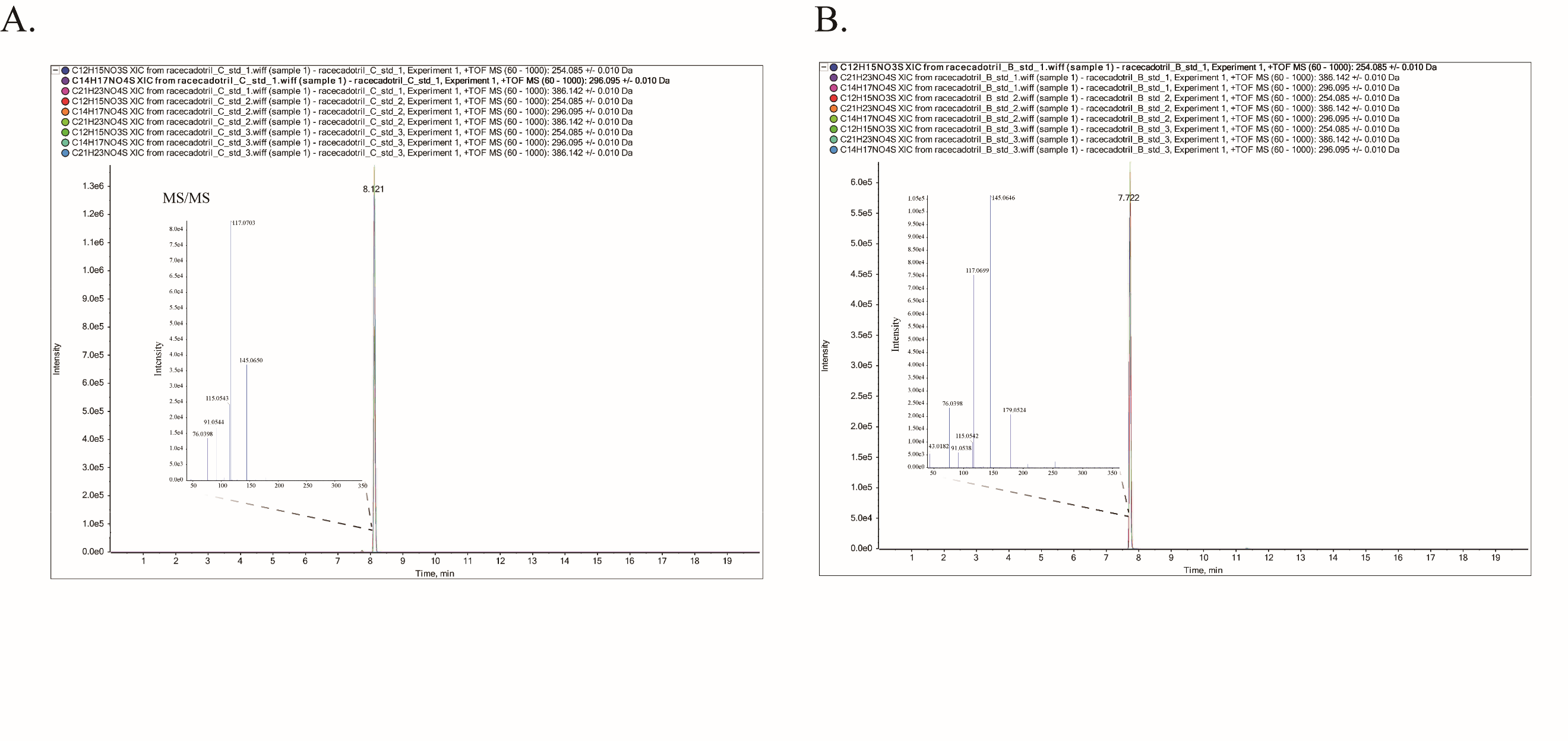


**Figure S8** Non-targeted metabolomics analysis of reference standards. The overlay total extracted ion chromatograms (XICs), retention time, and tandem mass (MS2) spectra of (A) S-acetylthiorphan and (B) thiorphan.


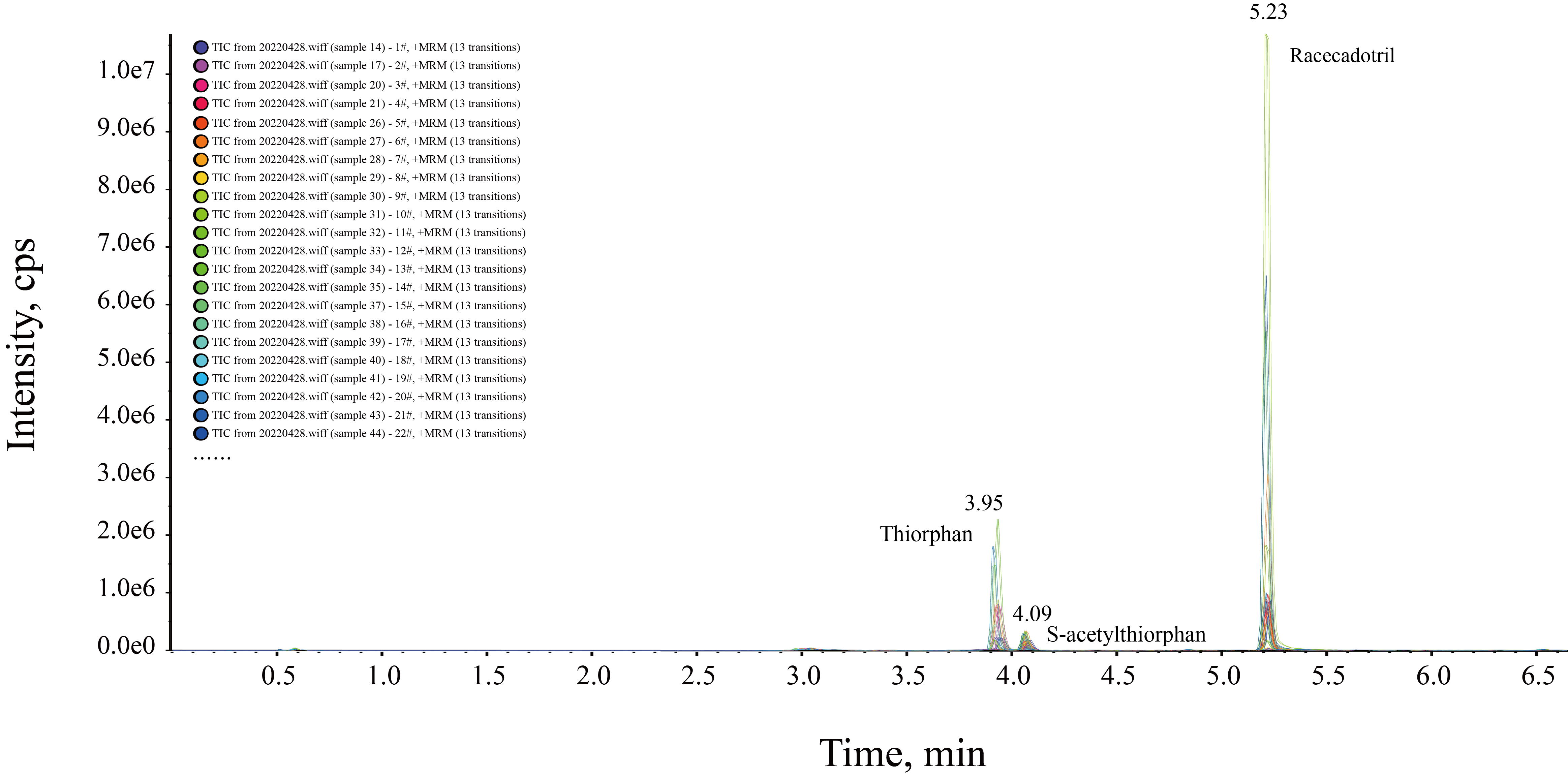


**Figure S9** The overlay total ion chromatogram (TIC) of individuals’ ex vivo fecal cultures incubated with racecadotril for 24 h at 37 ℃.

**
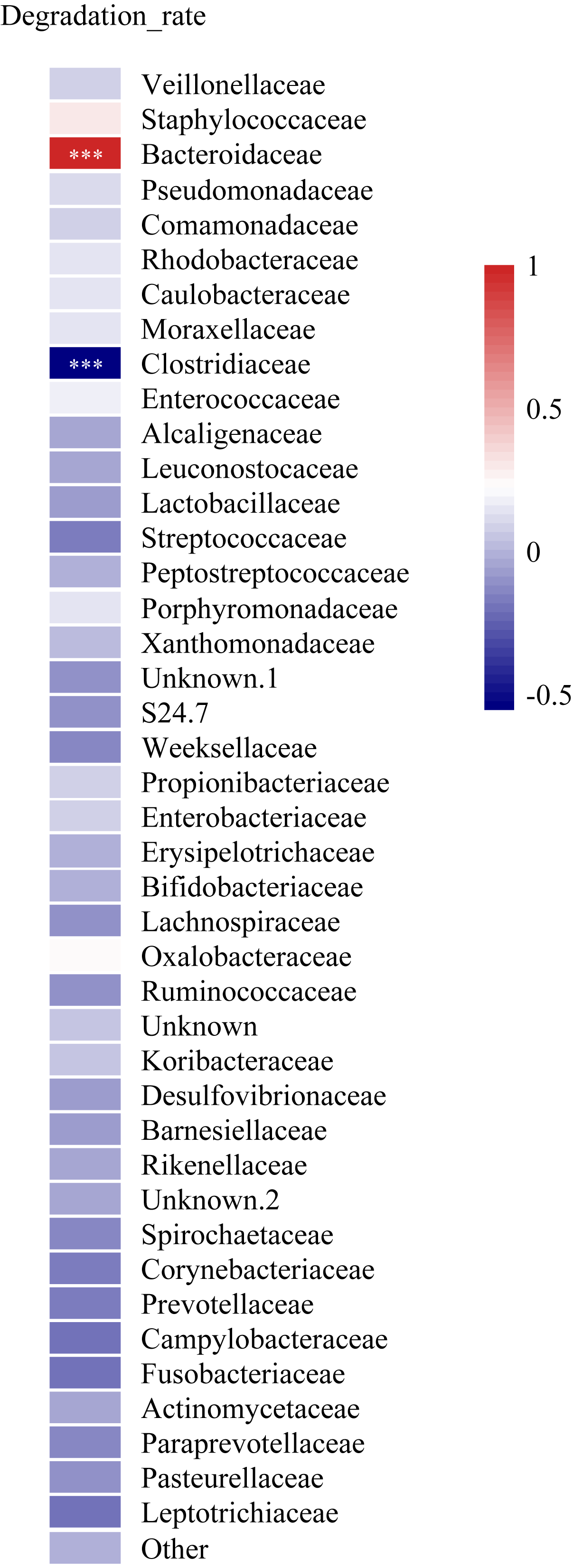
**

**Figure S10** Pearson’s correlation between the degradation rate of racecadotril and bacterial abundance. The bacterial community was annotated to the family level. The color scale shows the strength of correlation, ranging from 0 to 1 for positive correlation and from 0 to -0.5 for the negative correlation. A darker color intensity represents a stronger correlation and vice versa. *** *P* < 0.001.


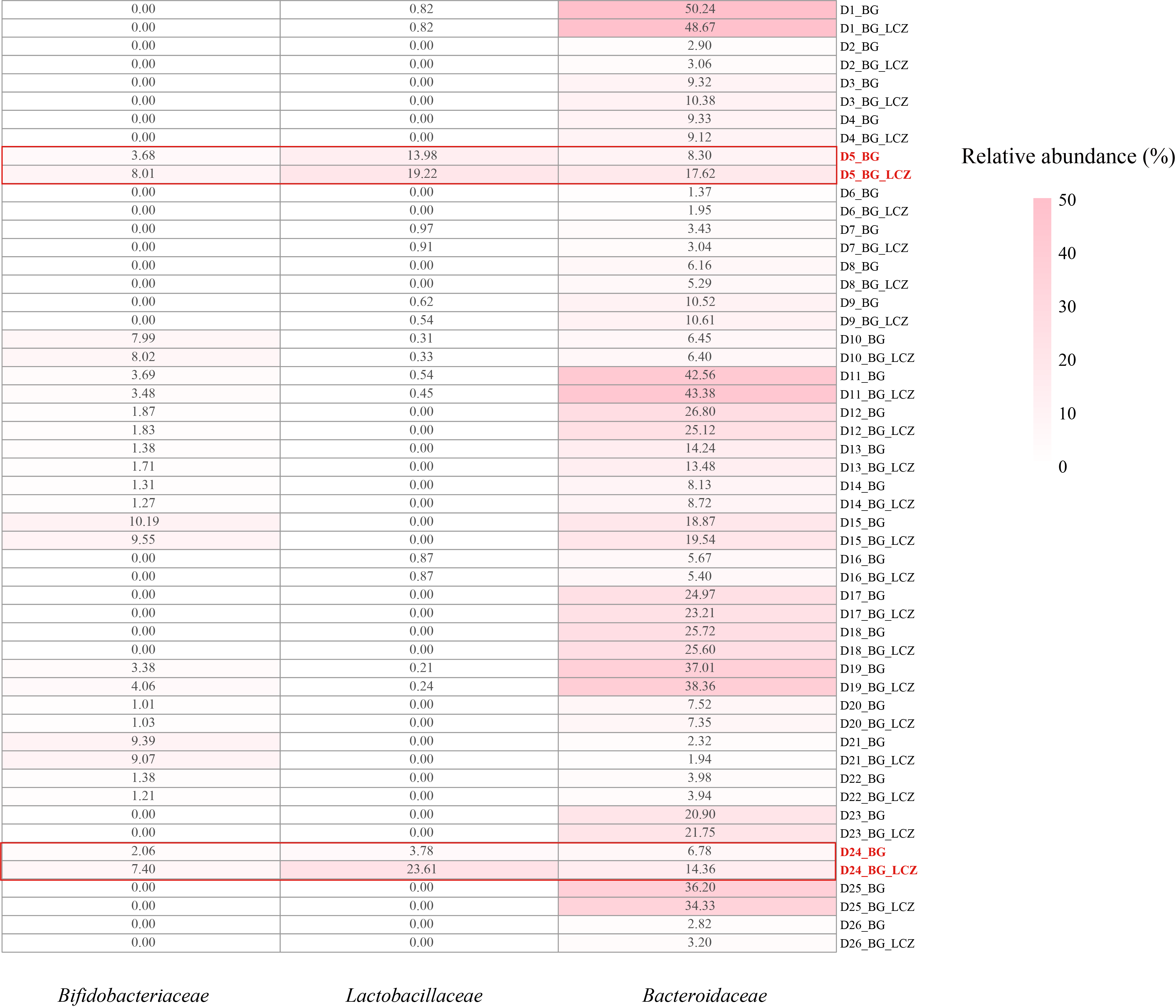


**Figure S11** The relative abundance of *Bacteroidaceae*, *Bifidobacteriaceae*, and *Lactobacillaceae* in ex vivo fecal cultures before and after incubation with racecadotril and LCZ for 24 hours.


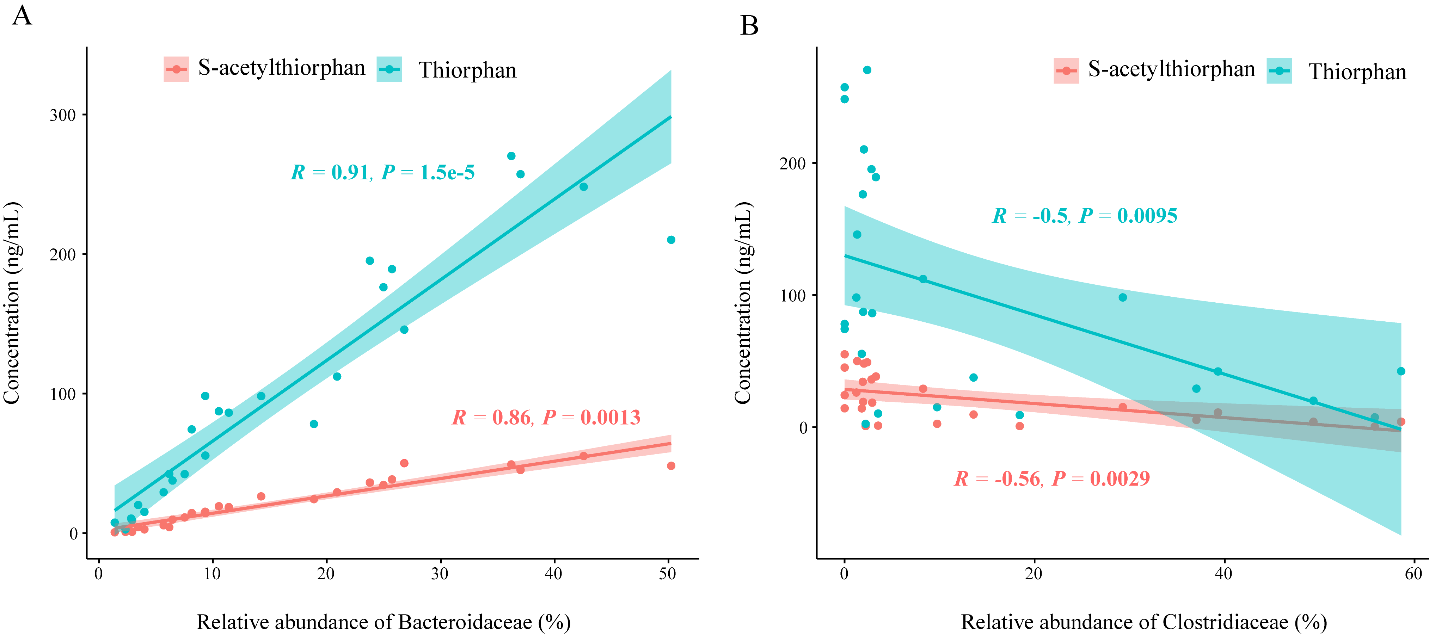


**Figure S12** Pearson’s correlation between the concentrations of thiorphan/S-acetylthiorphan and (A) *Bacteroidaceae*, (B) *Clostridiaceae*.
